# Macaque interferon-induced transmembrane proteins limit replication of SHIV strains in an Envelope-dependent manner

**DOI:** 10.1371/journal.ppat.1007925

**Published:** 2019-07-01

**Authors:** Amit Sharma, Richard N. McLaughlin, Ryan S. Basom, Caroline Kikawa, Molly OhAinle, Jacob S. Yount, Michael Emerman, Julie Overbaugh

**Affiliations:** 1 Human Biology Division, Fred Hutchinson Cancer Research Center, Seattle, WA, United States of America; 2 Pacific Northwest Research Institute, Seattle, WA, United States of America; 3 Genomics and Bioinformatics Shared Resource, Fred Hutchinson Cancer Research Center, Seattle, WA, United States of America; 4 Department of Microbial Infection & Immunity, The Ohio State University, Columbus, OH, United States of America; University of Wisconsin, UNITED STATES

## Abstract

HIV-1 does not persistently infect macaques due in part to restriction by several macaque host factors. This has been partially circumvented by generating chimeric SIV/HIV-1 viruses (SHIVs) that encode SIV antagonist of known restriction factors. However, most SHIVs replicate poorly in macaques unless they are further adapted in culture and/or macaques (adapted SHIVs). Therefore, development of SHIVs encoding HIV-1 sequences derived directly from infected humans without adaptation (unadapted SHIVs) has been challenging. In contrast to the adapted SHIVs, the unadapted SHIVs have lower replication kinetics in macaque lymphocytes and are sensitive to type-1 interferon (IFN). The HIV-1 *Envelope (Env)* in the chimeric virus determines both the reduced replication and the IFN-sensitivity differences. There is limited information on macaque restriction factors that specifically limit replication of the more biologically relevant, unadapted SHIV variants. In order to identify the IFN-induced host factor(s) that could contribute to the inhibition of SHIVs in macaque lymphocytes, we measured IFN-induced gene expression in immortalized pig-tailed macaque (Ptm) lymphocytes using RNA-Seq. We found 147 genes that were significantly upregulated upon IFN treatment in Ptm lymphocytes and 31/147 were identified as genes that encode transmembrane helices and thus are likely present in membranes where interaction with viral Env is plausible. Within this group of upregulated genes with putative membrane-localized proteins, we identified several interferon-induced transmembrane protein (IFITM) genes, including several previously uncharacterized Ptm *IFITM3*-related genes. An evolutionary genomic analysis of these genes suggests the genes are *IFITM3* duplications not found in humans that are both within the *IFITM* locus and also dispersed elsewhere in the Ptm genome. We observed that Ptm IFITMs are generally packaged at higher levels in unadapted SHIVs when compared to adapted SHIVs. CRISPR/Cas9-mediated knockout of Ptm *IFITMs* showed that depletion of IFITMs partially rescues the IFN sensitivity of unadapted SHIV. Moreover, we found that the depletion of IFITMs also increased replication of unadapted SHIV in the absence of IFN treatment, suggesting that Ptm IFITMs are likely important host factors that limit replication of unadapted SHIVs. In conclusion, this study shows that Ptm IFITMs selectively restrict replication of unadapted SHIVs. These findings suggest that restriction factors including IFITMs vary in their potency against different SHIV variants and may play a role in selecting for viruses that adapt to species-specific restriction factors.

## Author summary

Macaque model systems are critical gatekeepers for testing HIV-1 prevention methods and for studies of HIV-1 transmission and pathogenesis. HIV-1 does not persistently infect macaques due to inhibition of the virus by several macaque-specific restriction factors necessitating the use of chimeric SIV/HIV-1 viruses (SHIVs). Existing SHIV/macaque models typically employ SHIVs that encode HIV-1 sequences from viruses amplified in culture and further adapted in macaques (adapted SHIVs). Development of SHIVs encoding circulating HIV-1 variants derived directly from infected humans (unadapted SHIVs), which best model HIV-1 infection in humans, has been challenging as these SHIVs replicate poorly in macaque cells. While some host restrictions to HIV-1 replication in macaques have been defined, there is limited information on macaque-specific restriction factors that limit replication of circulating HIV-1 variants. Here, we demonstrate that the macaque interferon-induced transmembrane proteins (IFITMs) selectively restrict replication of unadapted SHIVs. These findings may help develop new approaches to improve the SHIV/macaque model of HIV-1 infection by rationally designing clinically-relevant SHIVs that overcome restriction by macaque-specific restriction factors.

## Introduction

The macaque models of HIV-1 infection have been critical to the understanding of retroviral pathogenesis as well as for testing antiretroviral therapies and candidate vaccines for HIV-1. However, multiple species-specific host factors restrict HIV-1 replication in macaque cells [1]. To overcome these restrictions chimeric SIV/HIV-1 viruses (SHIVs), which encode the SIV antagonists of known restriction factors are used to infect macaques to model HIV-1 infection. Existing SHIV/macaque models typically employ SHIVs that encode HIV-1 sequences from viruses that were adapted by viral passage in cell culture, and often these viruses are from chronic stages of infection. However, there is evidence that the chronic stage HIV-1 variants are distinct from HIV-1 variants that are selected for transmission in humans [2, 3]. In addition, SHIVs encoding HIV-1 sequences derived directly from humans typically require further adaptation *in vitro* in macaque cells and/or *in vivo* by serial macaque-passage [1] in order to obtain pathogenic viruses that establish persistent infection in macaques. These variant chimeric viruses used in the SHIV/macaque models are thus “adapted” SHIVs. We have previously determined that most circulating, transmitted HIV-1 Envelope (Env) variants, including the transmitted/founder variants, do not use macaque CD4 entry receptor efficiently [4] and thus SHIVs generated using these Envs replicate poorly in macaque cells. The adaptation of SHIV *Env* sequences in macaques increases replication and pathogenicity of SHIVs [5–11] but also leads to antigenic changes in Env that can limit their utility for vaccine and therapeutic approaches [12]. SHIVs encoding circulating HIV-1 variants obtained directly from the newly infected patients without adaptation (termed unadapted SHIVs) that maintain the antigenic properties of the transmitted variants are desired as challenge viruses for vaccine and therapeutic studies. However, most attempts at generating these SHIVs have failed as unadapted SHIVs replicate poorly, if at all, in macaque cells and do not establish persistent infection [13]. The virus-host dynamics that contribute to the differences in replication of adapted and unadapted SHIVs in macaques are not well defined.

One of the main host determinants that exerts immune pressure on viruses *in vivo* is the type-1 interferon (IFN) response. Upon infection, detection of viral pathogen-associated molecular patterns (PAMPs) by host pattern recognition receptors (PRRs) activates a signaling cascade that results in production of IFNα/β and other inflammatory cytokines [14]. IFNα/β in turn activates autocrine or paracrine IFN-signaling pathways that results in expression of hundreds of IFN-stimulated genes (ISGs)–thus inducing an “antiviral state”. Proteins encoded by certain ISGs such as APOBEC3s, TRIM5, Tetherin/BST-2, and MxB/Mx2 potently block lentiviral replication [15, 16] and are referred to as “restriction factors”. Lentiviruses have in turn evolved evasion/escape mechanisms and encode viral antagonists to counteract the restriction factors. These antagonistic virus-host interactions have resulted in an evolutionary “arms race” that drives continuous rounds of selection for advantageous mutations in the restriction factor genes [17]. Due to viral-host coevolution and because viruses can evolve faster than their hosts, lentiviral antagonists in turn adapt to the restriction factors encoded by their natural hosts. Thus, restriction factors are less active against wild-type viruses replicating in their natural host but act as potent barriers against cross-species transmission [15, 16].

HIV-1 infection in humans and SIV infection in macaques induces a robust IFN response during acute infection *in vivo* [16, 18–20]. Despite the induction of an IFN response, HIV-1 and SIV replication persists in their respective natural hosts. Recent studies suggest that transmitted strains of HIV-1 are more IFN-resistant than strains obtained from chronic stages of infection [21, 22], although this has not been seen in all studies [23, 24]. However, in the case of unadapted SHIV infection in macaques, the selection pressure may be distinct as the HIV-1 portion of the genome has been adapted in humans and SHIVs therefore may be under a different selection pressure that is more similar to a cross-species transmission event. In some cases, adapted rapidly replicating, pathogenic SHIVs have been derived by rapid serial macaque-passage, performed within the first two weeks of infection, a time during which macaques mount a robust IFN response to infection [18, 20], potentially implicating escape from the IFN response as contributing to the adaptation process.

Recently, we examined the sensitivity of a panel of adapted and unadapted SHIVs to IFNα in macaque lymphocytes and found that the unadapted SHIVs were potently inhibited by IFNα despite encoding the SIV antagonists of known restriction factors [25]. In contrast, SHIVs encoding adapted HIV-1 variants were largely resistant to IFNα. This difference mapped to HIV-1 *Env*, which was also a major determinant of replication differences, with adapted SHIVs demonstrating more rapid replication kinetics than unadapted SHIVs in macaque lymphocytes. These findings suggest that there are macaque-specific ISGs that restrict replication of unadapted SHIVs, and the adaptation of *Env* potentially plays a role in evading or antagonizing the macaque IFN response.

The goals of this study were to characterize the host IFN response in macaque lymphocytes and to identify IFN-induced host factor(s) that specifically inhibit replication of unadapted SHIVs. To this end, we measure the IFN-induced gene expression in immortalized pig-tailed macaque (Ptm) CD4+ lymphocytes and identify previously uncharacterized interferon-induced transmembrane protein (IFITM) genes as candidate host factors for the observed IFN inhibition of unadapted SHIVs. The IFITM family of proteins are small (125–135 amino acids) transmembrane proteins with a type II transmembrane protein topology that are expressed at a basal level in multiple cell types [26, 27]. Of the five *IFITM* genes encoded by humans, *IFITM1*, *IFITM2*, and *IFITM3* are induced by IFN and display broad antiviral activity against a number of enveloped viruses [28, 29]. Recent studies have shown that human IFITMs (IFITM1, 2 and 3) are incorporated into HIV-1 virions and impair viral fusion and cell-to-cell spread [30–33]. Here, we demonstrate that Ptm IFITMs are packaged at higher levels in unadapted SHIVs when compared to adapted SHIVs, that depletion of IFITMs partially rescues the IFN sensitivity of unadapted SHIVs, and that depletion of basal levels of IFITMs increases replication of unadapted SHIVs in the absence of IFN. These findings identify IFITMs as IFN-induced host factors that limit replication of SHIVs in macaque lymphocytes in an Env-dependent manner.

## Results

### IFNα-induced gene expression in macaque lymphocytes

**Fig 1:**
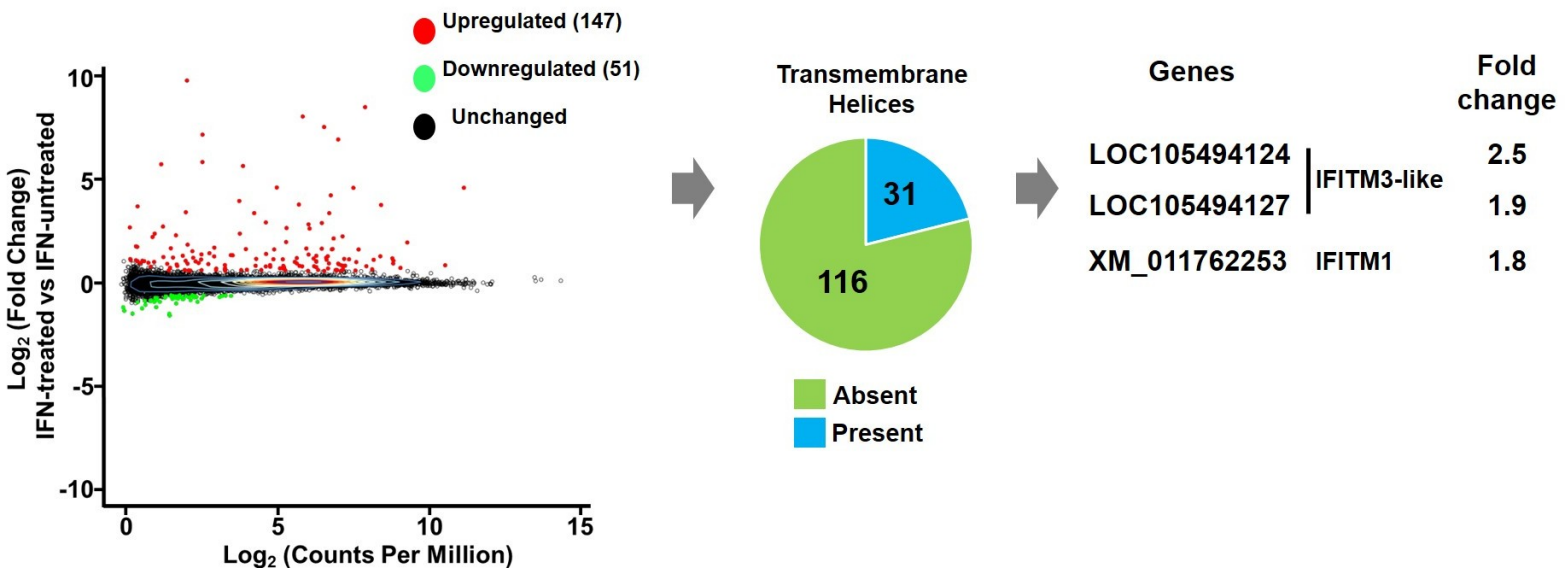
Identification of IFNα-stimulated genes (ISGs) in macaque lymphocytes. Dot plot of log_2_ of average expression (x axis) per log_2_ of fold change (y axis) for all genes identified upon IFNα treatment (1000 U/ml for 24 hours) of Ptm lymphocytes. 198 genes that were differentially expressed (|logFC| ≥ 0.585 & FDR 5%) are highlighted: the 147 genes that were significantly upregulated are shown with red dots and the 51 genes that were significantly downregulated are shown with green dots. The pie chart shows the fraction of genes that contain transmembrane helices based on translation of open reading frames of the upregulated genes. The fold change of the indicated Ptm *IFITM3-like* and *IFITM1* that were selected for subsequent analysis are shown.

In order to characterize the IFN response in macaque lymphocytes and to identify IFNα-induced host factor(s) that might inhibit replication of unadapted SHIVs, we measured IFNα-induced gene expression in immortalized Ptm CD4+ lymphocytes. Triplicate cultures of Ptm lymphocytes were left untreated or treated with IFNα at a concentration similar to that observed in natural SIV and HIV-1 infection (1000 U/ml) [18, 20, 34]. Twenty-four hours later, RNA was isolated, and RNA-seq libraries were prepared for sequencing. Comparable numbers of reads were obtained for each sample (average ~21.5 million reads for untreated and ~21.2 million reads for IFNα-treated, respectively) and similar percentages of reads were unambiguously mapped (average ~65.3% for untreated and ~64.9% for IFNα-treated, respectively) to the pig-tailed macaque genome (*M*. *nemestrina* Mnem 1.0). A total of 198 genes were found to be significantly differentially expressed (|logFC| ≥ 0.585 & FDR 5%) upon IFNα treatment (Fig 1). 147/198 genes were found to be significantly upregulated and 51/198 genes were found to be significantly downregulated upon IFNα treatment (Fig 1, S1 Table).

In order to identify candidate IFNα-induced host factor(s) that inhibit replication of unadapted SHIVs, we prioritized the upregulated genes and applied the following criteria. Because the differences in IFNα-induced inhibition between SHIVs mapped to Env we prioritized upregulated genes with predicted subcellular localization at sites of viral entry or assembly, such as intracellular and plasma membranes where interaction with Env is plausible. For this, we determined the amino acid sequences of the open reading frames (ORFs) of the 147 significantly upregulated genes and used TMHMM v2.0 [35] to predict which of these encode transmembrane helices. We found that 31/147 genes were predicted to encode at least one transmembrane helix (Fig 1, S2 Table). Based on these criteria of differential expression upon IFNα treatment and predicted membrane localization, we selected two previously uncharacterized Ptm genes (LOC105494124 and LOC105494127: both predicted to be IFITM3-like genes) and IFITM1 (XM_011762253) for subsequent analysis (Fig 1). Notably, IFITM1 and IFITM3 from other species have been reported as broad-spectrum, anti-viral factors that impair viral fusion [28, 29].

### Characterization of macaque *IFITMs*

**Fig 2:**
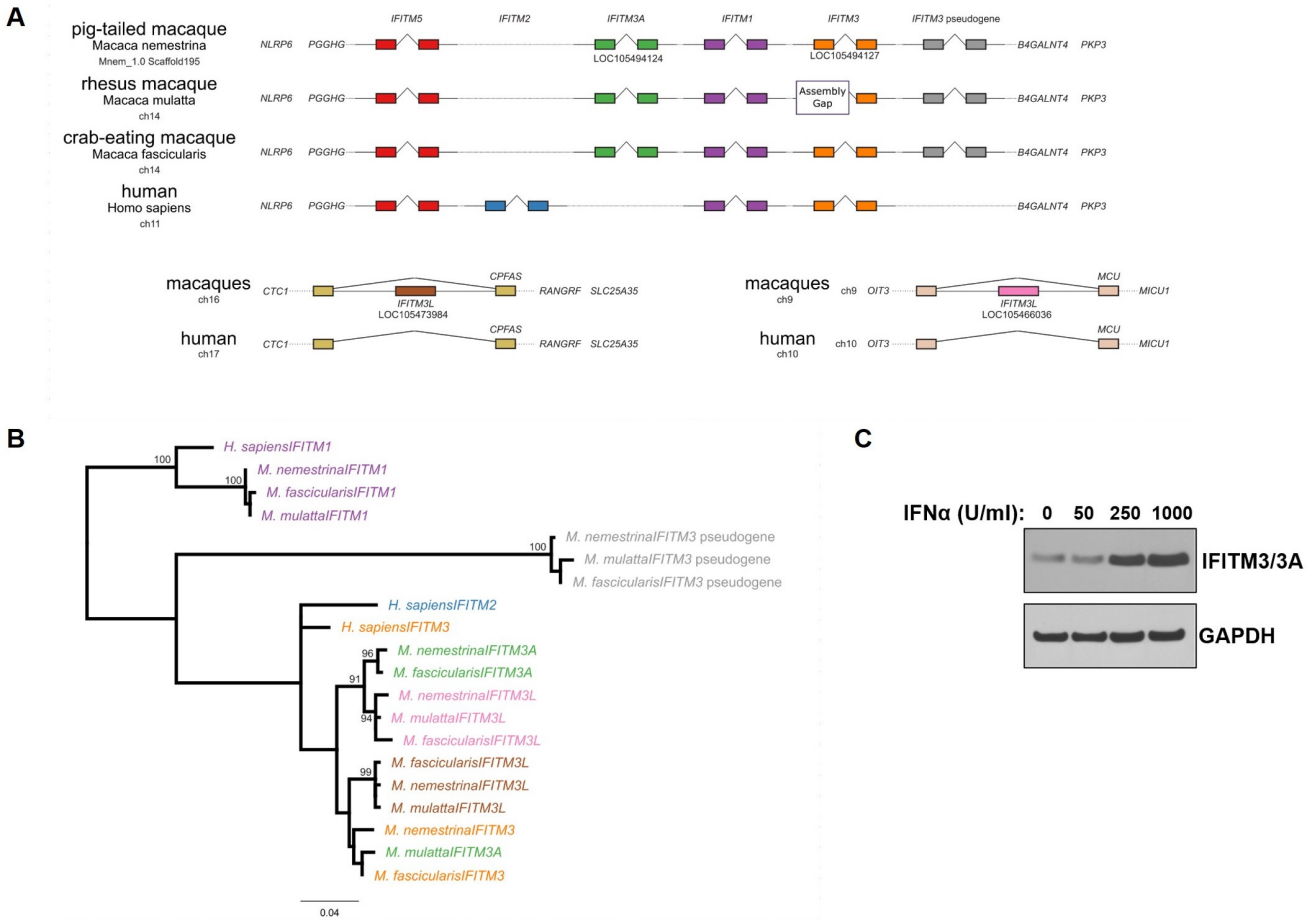
Characterization of macaque *IFITMs*. **(A)** A comparison of the chromosomal context of *IFITM* and *IFITM-like* genes in macaques and human. *Top panel*: Comparison of the human *IFITM* locus on chromosome 11 (abbreviated “ch11”) of the human reference genome and the corresponding loci in the indicated macaque genomes (chromosome 14 “ch14” for rhesus and crab-eating macaques, and Mnem_1.0 Scaffold195 for pig-tailed macaque). Exons are indicated as filled rectangles and introns as joined angled lines. Gene names are indicated in italics. Vertical alignment of conserved flanking genes *NLRP6*, *PGGHG*, *B4GALNT4*, and *PKP3* across species is indicative of synteny. Humans encode *IFITM2* (blue), a duplication of *IFITM3*, which is not present in the macaque genomes. The parental *IFITM3* (orange, LOC105494127 in pig-tailed macaque) is present in human and macaques. Macaques genomes contain an additional copy of *IFITM3* (indicated *IFITM3A*, LOC105494124, green) and an *IFITM3* pseudogene (gray). *Bottom panel*: Two additional *IFITM3*-like genes identified in macaques in different chromosomal contexts. One *IFITM3*-like gene (indicated *IFITM3L*, LOC105473984, brown) is located in the intron of *CPFAS* on chromosome 16 (ch16). The other gene (indicated *IFITM3L*, LOC105466036, pink) is located in the intron of *MCU* on chromosome 9 (ch9). The corresponding loci in the human genome are indicated below and lack *IFITM3*-like genes. **(B)** A maximum likelihood tree of *IFITM* nucleotide sequences shows groupings of *IFITM1*, *IFITM3* pseudogenes, and *IFITM3/2*. Tree is rooted on the *IFITM1* group. Text colors correspond to rectangles depicting the same sequences in panel A. Nodes with bootstrap support greater than 90 are labeled. **(C**) Western blot analysis of Ptm lymphocytes treated with increasing concentrations of IFNα for 24 hours. The concentration of IFNα is indicated above each lane. Immunoblotting performed using anti-IFITM3/3A and anti-GAPDH antibodies.

Previous work to determine the number and location of *IFITM* sequences within animal genomes focused broadly on vertebrates [36] or primates [37]. As a result, variation in IFITM genes amongst closely related species remains largely undescribed. Since our RNA-seq analysis showed increased expression of multiple Ptm IFITM-related genes, including some uncharacterized sequences, we wanted to understand the location, sequence, and evolutionary relationship of previously described human IFITMs and the IFNα-upregulated Ptm IFITMs (Fig 1). We mapped the relative position of all *IFITM* genes within the canonical *IFITM* locus, focused on comparing the genome of three macaques (pig-tailed, rhesus, and crab-eating) with human. The macaque *IFITM* loci were mapped using the human *IFITM1*, *IFITM3*, and *IFITM5* sequences as queries of each macaque genome assembly. *IFITM1* and *IFITM5* had a single clear hit in each genome as compared to the human locus, suggesting one-to-one orthology of these genes (Fig 2A; top panel; purple and red). In contrast, *IFITM3* mapped to three unique locations within the *IFITM* locus of each macaque genome. An analysis of the synteny of these sequences across macaques and humans shows that one of these sequences is syntenic with human *IFITM3* (Fig 2A; top panel; orange), while the other two sequences are not found in humans. Of these macaque *IFITM3* sequences, two encode apparently intact IFITM3 genes (Fig 2A; top panel; green and orange), while the third copy has been pseudogenized with mutations that introduce numerous stop codons (Fig 2A; top panel; gray).

Humans have a duplication of *IFITM3*, named *IFITM2* [36], and a phylogenetic tree shows that all the intact and pseudogenized macaque *IFITM3* genes group together with the human *IFITM2* and *IFITM3* sequences (Fig 2B and S1 File). While *IFITM2* has only been described in humans, chimpanzees, and gorillas [37], we find that macaques also have a duplication of *IFITM3* within the *IFITM* locus. This copy LOC105494124, here called *IFITM3A*, retains an intron and an ORF of the same length (~402nt) as the parental IFITM3 (LOC105494127).

In addition to the IFITM3 sequences within the IFITM locus of the macaques, we found numerous shorter sequences (100-400bp) outside of the *IFITM* locus with sequence identity to *IFITM3* greater than 80%. These hits represent partial and full-length copies of *IFITM* genes. Two of these sequences were identified in the RNA-seq analysis, denoted as *IFITM3L* sequences on chromosomes 16 and 9 (Fig 2A; bottom panel; brown, pink). These copies are both found in the intronic region of genes and have lost the intron present in *IFITM* sequences within the *IFITM* locus. Based upon their phylogenetic grouping with IFITM3, their dispersion in the genome, and their lack of introns, these sequences are likely retrocopies of *IFITM3*, often referred to as pseudogenes of *IFITM3*. However, both of these retrocopies preserve a putative ORF similar in length to *IFITM3* and we find these copies to be expressed.

We compared the magnitude of IFNα-dependent induction of these IFITM-genes by mapping the reads from our RNA-seq data to the Ptm genomic loci. We found that the bulk of the RNA-seq read coverage for IFITM3A and IFITM1 was from uniquely mapping reads (S1 Fig). For IFITM3, there were a considerable number of ambiguous reads that did not map uniquely, particularly in the first exon (S1 Fig). However, there was still enough coverage from the uniquely mapping reads to suggest that this locus was expressed at the RNA level. In addition, we measured the levels of IFITM protein expression upon IFNα treatment in Ptm lymphocytes. Out of five commercially available IFITM antibodies tested, we found two that react with Ptm IFITMs and selected the one with the highest reactivity for subsequent analysis. Importantly, the selected anti-human IFITM3 antibody used in this study does not distinguish between Ptm IFITM3 and Ptm IFITM3A (S2 Fig); therefore, the immunoblots using this antibody are indicated as “IFITM3/3A”. Our immunoblotting results indicate that IFITM3/3A is induced by IFNα in a dose dependent manner in Ptm lymphocytes (Fig 2C).

Lastly, we performed immunofluorescence imaging of the Ptm IFITMs in mouse embryonic fibroblasts (MEFs) lacking the IFITM locus (IFITMdel) [38] and HEK293T cells, which also lack baseline IFITM expression [39]. In both cell types, the IFITMs were observed in intracellular clusters primarily in the perinuclear region, and IFITM1 staining was also observed at the plasma membrane (S3 Fig). The cellular localization of Ptm IFITMs is similar to what is reported for human IFITMs [30, 40].

### Incorporation of macaque IFITMs in SHIV virions

Incorporation of human IFITMs (IFITM1, 2 and 3) into HIV-1 virions impairs viral fusion and cell-to-cell spread [30–33]. In addition, human IFITMs affect HIV-1 Env processing and virion Env incorporation [41]. Thus, we explored whether Ptm IFITMs contribute to the sensitivity of SHIVs to IFNα treatment in macaque lymphocytes and whether they explain Env-dependent differences in IFNα sensitivity between adapted and unadapted SHIVs.

**Fig 3:**
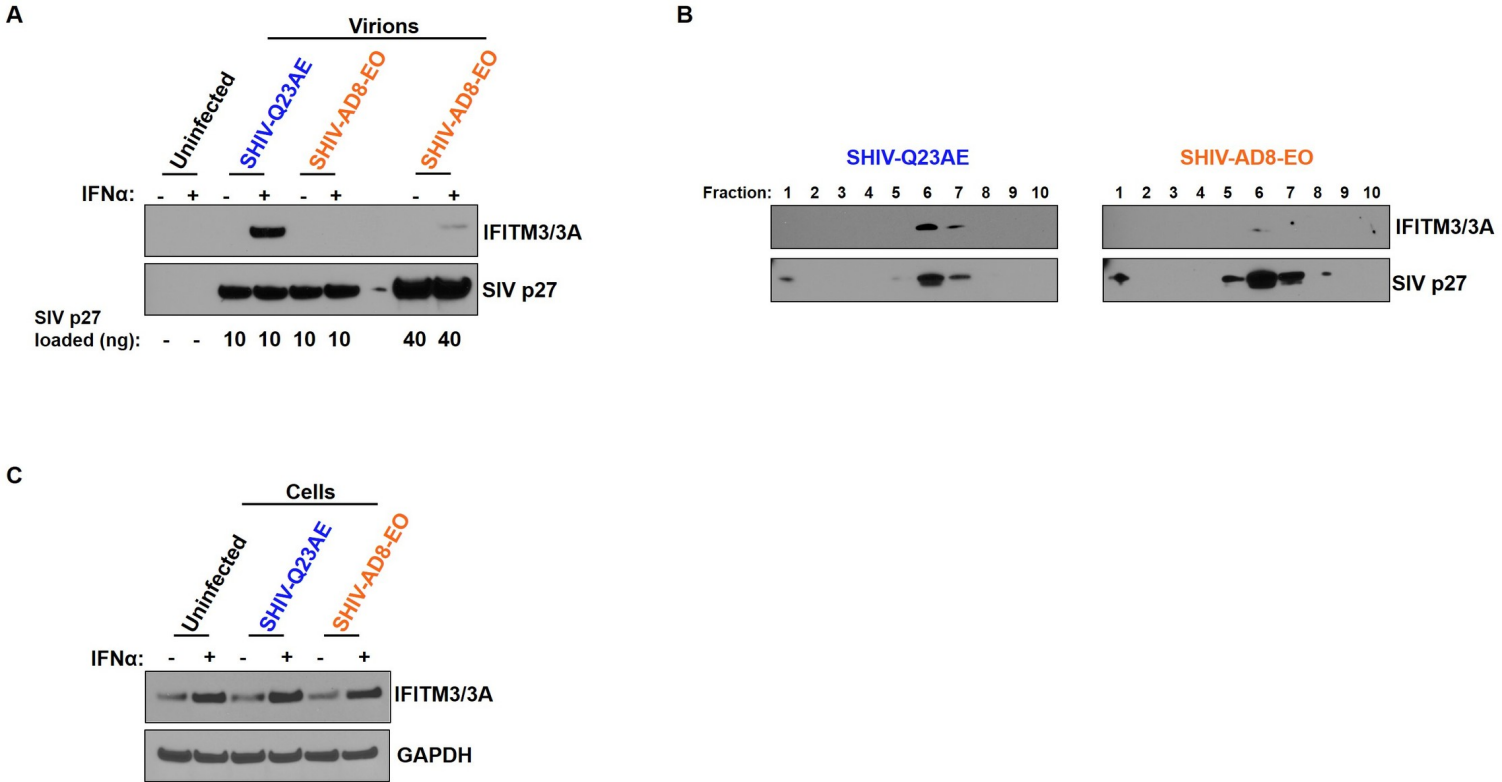
Incorporation of macaque IFITM3 in SHIV virions. **(A)** Western blot analysis of virions harvested at nine days post-infection from Ptm lymphocytes infected with indicated SHIV. Color-coding indicates whether the SHIV is adapted (orange) or unadapted (blue). Virions produced in the absence or presence of IFNα (1000 U/ml) is indicated above each lane. The values below the bottom panel indicate the amount of virions (ng of SIV p27) loaded into each lane. **(B)** Western blot analysis of virions purified using sucrose density gradient. The numbers above each lane indicate the fraction collected from the top of the gradient. **(C)** Western blot analysis of Ptm lymphocytes infected with indicated SHIV and harvested at nine days post-infection. Immunoblotting performed using anti-IFITM3/3A, anti-SIV p27, and anti-GAPDH antibodies.

We employed a prototypical, macaque-adapted SHIV (SHIV AD8-EO) and a prototypical, unadapted SHIV (SHIV Q23AE). SHIV AD8-EO was derived by five serial-macaque passages followed by further adaption in macaque PBMCs [8] while SHIV Q23AE was generated from a HIV-1 Env from early in infection and then modified with a single amino acid substitution (A204E) to allow the Env to use macaque CD4 for entry [42]. In order to determine whether or not the Ptm IFITMs are differentially incorporated into unadapted versus adapted SHIV virions, Ptm lymphocytes were infected with SHIV AD8-EO and SHIV Q23AE. Virions were harvested nine days post-infection and an amount of virus equivalent to 10 ng of SIV p27 were immunoblotted. We found that Ptm IFITM3/3A was readily detected in the unadapted SHIV Q23AE virions but not in the adapted SHIV AD8-EO (Fig 3A). When four times more virions (40 ng of SIV p27) were immunoblotted, IFITM3/3A could be detected, but the level of IFITM3/3A in the adapted SHIV AD8-EO virions was lower than the unadapted SHIV Q23AE (Fig 3A). Virions were also purified by analytical sucrose density gradient fractionation to remove secreted, soluble cellular proteins and budding cellular microvesicles. Immunoblot analyses of the gradient fractions with IFITM3/3A and SIV p27 antibodies revealed co-fractionation of IFITM3/3A with SIV p27 suggesting that IFITM3/3A are incorporated in the SHIV virion (Fig 3B). Consistent with the results in Fig 3A, we observed that IFITM3/3A is packaged at higher levels in the unadapted SHIV Q23AE virions in comparison to the adapted SHIV AD8-EO virions. Importantly, the basal and IFNα-induced levels of IFITM3/3A were similar in uninfected cells or cells infected with adapted SHIV A8-EO or the unadapted SHIV Q23AE (Fig 3C). These findings suggest that infection with SHIV does not affect steady state or IFNα-induced IFITM levels in Ptm lymphocytes, but rather the Ptm IFITMs are differentially incorporated into an unadapted SHIV relative to an adapted SHIV.

**Fig 4:**
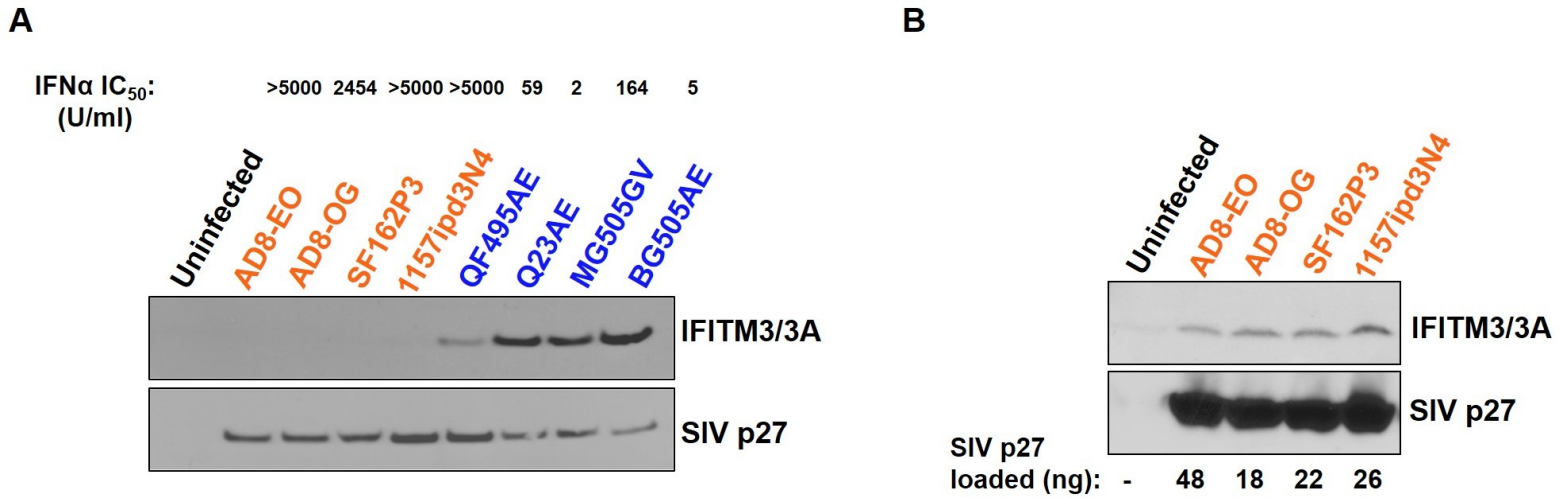
Differences in incorporation of macaque IFITM3 in SHIV virions. **(A)** Western blot analysis of virions harvested at nine days post-infection from Ptm lymphocytes infected with indicated SHIV in the presence of IFNα (1000 U/ml). Color-coding indicates whether the SHIVs are adapted (orange) or unadapted (blue). Virions equivalent to 5 ng of SIV p27 was loaded into each lane. IFNα sensitivity as measured by IFNα IC_50_ assay of each SHIV variant was previously determined [25] and is indicated at the top. **(B)** Western blot analysis of adapted SHIV virions. The values below the bottom panel indicate the amount of virions (ng of SIV p27) loaded into each lane. Immunoblotting performed using anti-IFITM3/3A and anti-SIV p27 antibodies.

Given the differences in IFITM packaging between the two prototype SHIVs, we determined whether the differences observed in IFITM incorporation are specific to the two SHIVs used in this study or also characteristic of other adapted and unadapted SHIVs. We employed a panel of eight SHIVs that we previously used to examine IFNα sensitivity in Ptm lymphocytes [25]. The panel includes four SHIVs encoding HIV-1 Env variants adapted through passage in cell culture and/or macaques (AD8-EO, AD8-OG, SF162P3, and 1157ipd3N4) and four SHIVs encoding HIV-1 Env variants isolated directly from infected individuals without culture or macaque adaption, three of which represent variants from early in infection (QF495AE, Q23AE, MG505GV, and BG505AE) and all of which encode a single amino acid substitution (A204E or G312V) to allow macaque CD4 use. The four unadapted SHIVs in this panel were potently inhibited by IFNα (IC_50_ range 2 to 164 U/ml) whereas the four adapted SHIVs were resistant to IFNα (IC_50_ range 2454 to >5000 U/ml) [25]. We utilized these SHIVs to examine virion incorporation of IFITMs. When virions equivalent to 5 ng of SIV p27 were immunoblotted, Ptm IFTIM3/3A was readily detected in the unadapted SHIV virions but not in the adapted SHIVs (Fig 4A). Thus, we observed that IFITM3/3A virion incorporation was linked to sensitivity to IFNα for this virus panel. We did detect some IFITM3/3A incorporation in adapted SHIV virions when 3.6- to 9.6-times more virions were loaded (Fig 4B) suggesting that IFITM3/3A is not totally excluded from these viruses. Taken together, our results suggest that Ptm IFITMs are packaged at higher levels in the unadapted SHIV virions in comparison to the adapted SHIV virions.

### Depletion of macaque IFITMs affect IFNα sensitivity of unadapted SHIV

In order to determine the contribution of Ptm IFITMs in restricting SHIV replication, we generated *IFITM*-knockout (KO) cell pools. We designed CRISPR guide RNA (crRNA) predicted to target Ptm *IFITM1*, *IFITM3A*, and *IFITM3* (Table 1). crRNA that did not target any macaque genes was used as a Non-Targeting Control (NTC). Because of the high sequence similarity between the Ptm IFITMs, one of our crRNA targeted both *IFITM1* and *IFITM3/3A* (this double KO is referred to as “M1+M3”).

**Table 1:** CRISPR guide RNA used in this study.

Gene name	Gene ID	Guide RNA sequence (5' - 3')	PAM sequence	Contig where gene is located	Target location of guide RNA	**Cut site**
IFITM1	XM_011762253	CUUGGGAGGAUGGUGCUAUG	GGG	NW_012011633.1	160219–160238	160235
IFITM3A	LOC105494124	UUGAGCAUCUCAUAGCUGGG	GGG	NW_012011633.1	148744–148763	148747
IFITM3	LOC105494127	GCACGAGGUGGCUAUGAUGG	GGG	NW_012011633.1	165106–165125	165109

**Fig 5:**
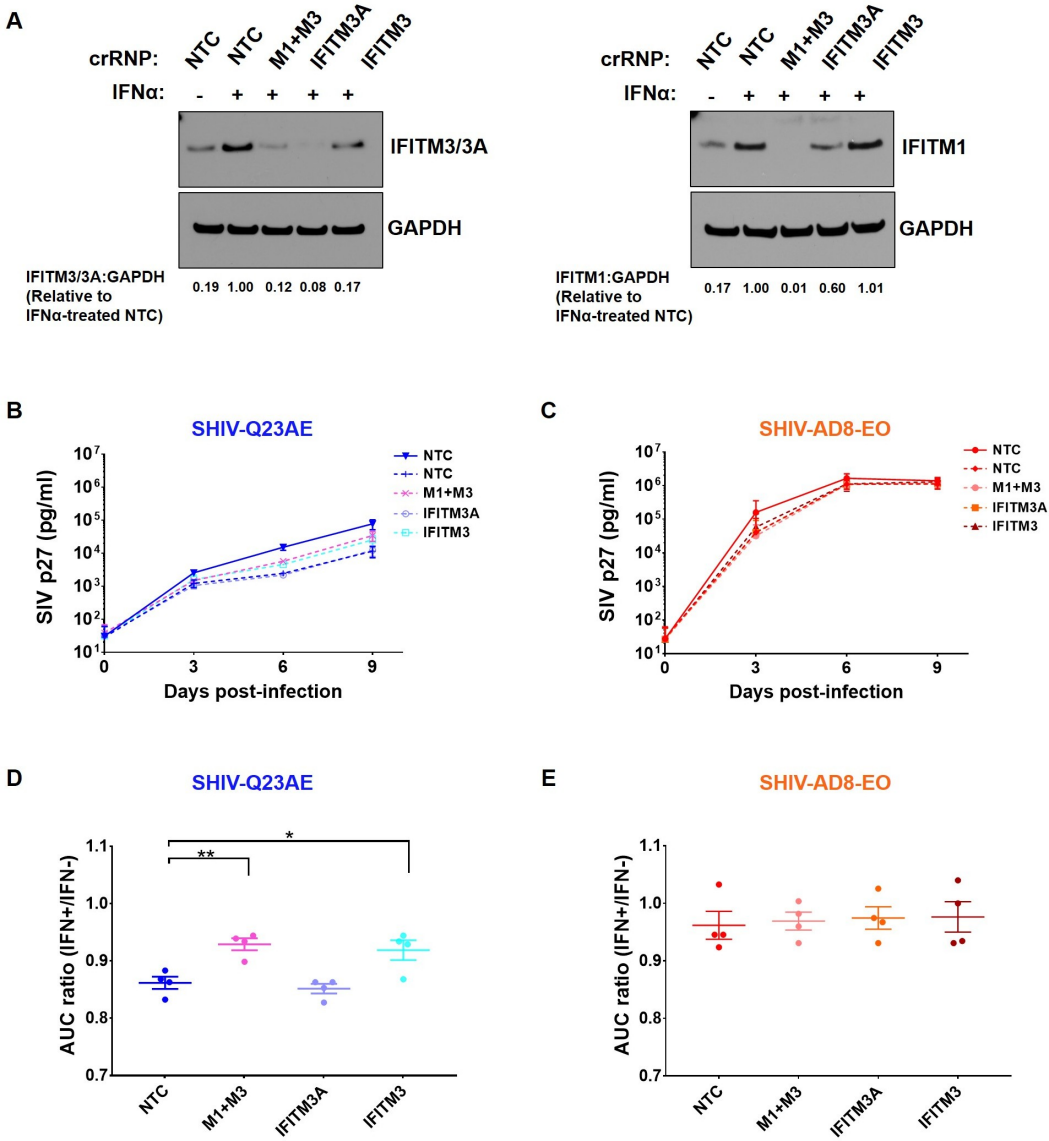
Knockout of macaque IFITMs affects IFNα sensitivity of unadapted SHIV. **(A)** Western blot analysis of Ptm *IFITM*-knockout cell pools using anti-IFITM3/3A (*left panel*) and anti-IFITM1 (*right panel*) antibodies. CRISPR/Cas9 ribonucleoproteins (crRNPs) that targeted *IFITM1* and *IFITM3* (indicated “M1+M3”), *IFITM3A*, *IFITM3*, or a non-targeting control (indicated “NTC”) is indicated at the top. IFNα-treatment (1000 U/ml) of cells is indicated above each lane. The numbers below the bottom panel indicate the IFITM3/3A:GAPDH (*left panel*) or IFITM1:GAPDH (*right panel*) signal relative to IFNα-treated, NTC. **(B–C)** Effect of IFNα-treatment on replication of SHIV-Q23AE **(B)** and SHIV-AD8-EO **(C)** in Ptm *IFITM*-knockout cell pools over a 9-day time course. The identity of each SHIV is indicated above the chart. Color-coding indicates whether the SHIVs are adapted (orange) or unadapted (blue). The key at the right of the graph indicates the color corresponding to each knockout cell line. Replication in the presence of IFNα (1000 U/ml) is indicated in the dashed lines, whereas control replication in the absence of IFNα is indicated in the solid lines. Data points represent the average of four independent experiments, and error bars represent SD. **(D–E)** Area under the curve (AUC) ratio for **(D)** SHIV-Q23AE and **(E)** SHIV-AD8-EO determined from the replication curves shown in (B) and (C), respectively. Data represent the average of four independent experiments, and error bars represent SEM. AUC values were compared using one-way analysis of variance (ANOVA) followed by Dunnett's multiple comparisons test. ** *p = 0*.*0054*, * *p = 0*.*0156*.

We generated four independent batches of *IFITM*-KO cell pools to examine whether IFITMs affect IFNα sensitivity of unadapted SHIV. IFNα-induced IFITM protein expression was confirmed through immunoblotting of IFNα-treated KO cells. The levels of IFITM3/3A in the IFNα-treated, KO cells were 5.9- to 12.5-fold lower compared to the IFNα-treated, NTC cells (Fig 5A; left panel; 8.3-fold for *M1+M3* KO, 12.5-fold for *IFITM3A* KO, and 5.9-fold for *IFITM3* KO), and comparable to control cells that were not treated with IFNα, suggesting a partial KO and/or presence of unedited cells in the KO pool. The levels of IFITM1 in the IFNα-treated, *M1+M3* KO cells were lower compared to the IFNα-treated (100 fold) or IFNα-untreated (17 fold) control cells (Fig 5A; right panel).

The KO cells were infected with the unadapted SHIV Q23AE or adapted SHIV AD8-EO at a multiplicity of infection (MOI) of 0.02 to allow spreading viral infection. The infected cells were cultured in the presence of 1000 U/ml of IFNα over a nine-day time course. Viral replication was also measured in the IFNα-untreated NTC cells to determine baseline replication. In order to obtain a quantifiable measure of IFNα sensitivity, we measured the ratio of the area under the curve (AUC) of the replication curve in the IFNα-treated cells to the AUC of the replication curve in the untreated, NTC cells. Consistent with our prior results, the prototype unadapted SHIV Q23AE exhibited a pronounced IFNα-induced inhibition of viral replication (6.7-fold at day 9 post infection) whereas the adapted SHIV AD8-EO was largely resistant to IFNα (AUC ratio 0.86 vs 0.96, *p = 0*.*0091 two-tailed student’s t-test*) (Fig 5B and 5C). Next, we compared the IFNα sensitivity of adapted and unadapted SHIV in *IFITM*-KO cells. We observed that in comparison to the NTC cells (AUC ratio 0.86), KO of *M1+M3* (AUC ratio 0.93, *p = 0*.*0054*) or *IFITM3* (AUC ratio 0.92, *p = 0*.*0156*), but not *IFITM3A* (AUC ratio 0.85), resulted in modest but statistically significant rescue of IFNα-induced inhibition of SHIV Q23AE replication (Fig 5B and 5D). In contrast, *IFITM* KOs had no significant effect on IFNα sensitivity of adapted SHIV AD8-EO (Fig 5C and 5E). Thus, the differential activity of IFITMs on the unadapted SHIVs accounts for some, but not all, of the IFNα sensitivity of these viruses.

### Depletion of macaque IFITMs increases replication of unadapted SHIV

**Fig 6:**
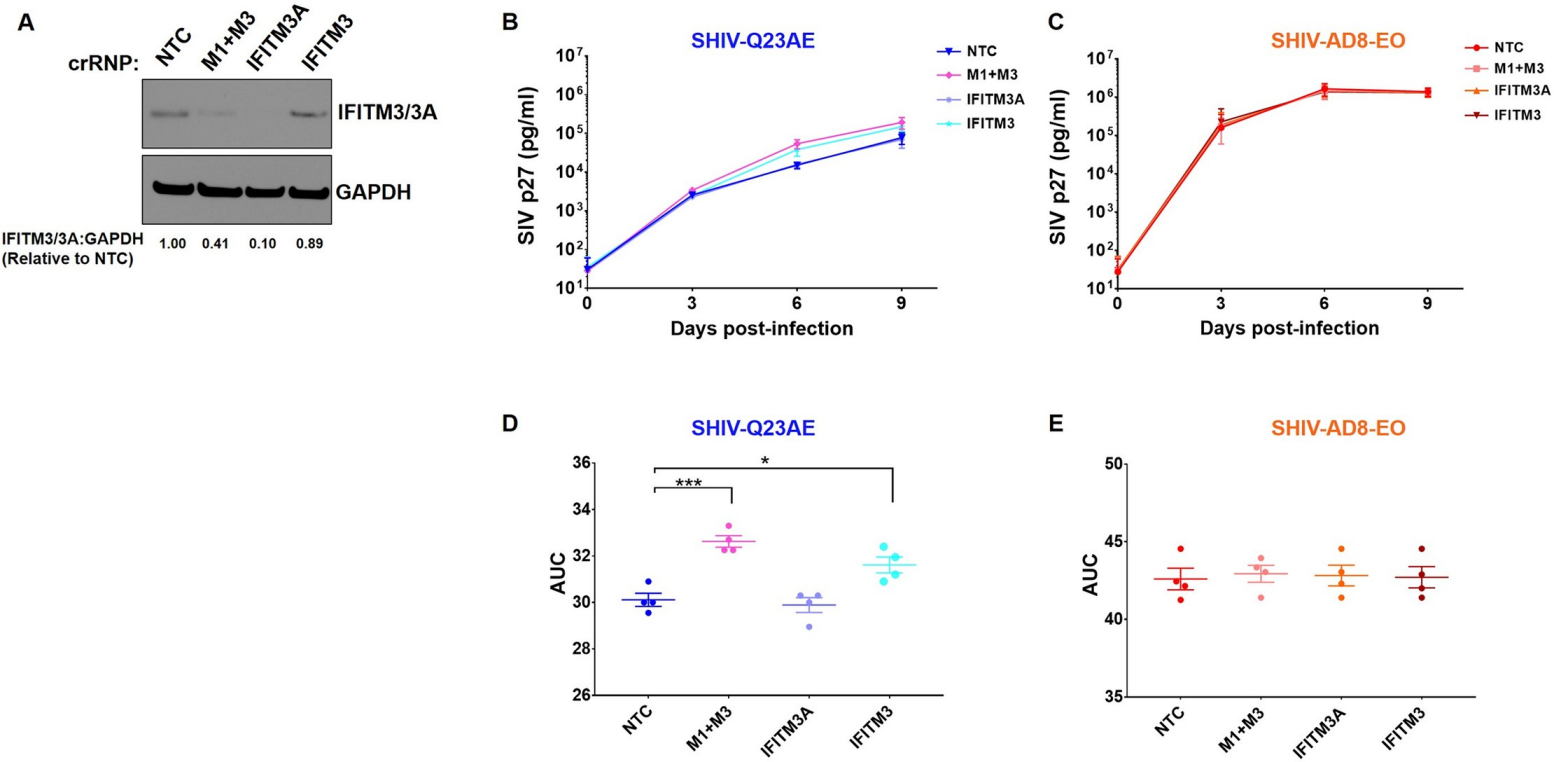
Knockout of macaque IFITMs increases replication of unadapted SHIV. **(A)** Western blot analysis of Ptm IFITM-knockout cell pools using anti-IFITM3/3A antibody. CRISPR/Cas9 ribonucleoproteins (crRNPs) that targeted *IFITM1* and *IFITM3* (indicated “M1+M3”), *IFITM3A*, *IFITM3*, or a non-targeting control (indicated “NTC”) is indicated at the top. The numbers below the bottom panel indicate the IFITM3/3A:GAPDH signal relative to NTC. **(B–C)** Replication kinetics of **(B)** SHIV-Q23AE and **(C)** SHIV-AD8-EO in Ptm *IFITM*-knockout cell pools over a 9-day time course. The identity of each SHIV is indicated above the chart. Color-coding indicates whether the SHIVs are adapted (orange) or unadapted (blue). The key at the right of the graph indicates the color corresponding to each knockout cell line. Data points represent the average of four independent experiments, and error bars represent SD. **(D–E)** Area under the curve (AUC) for **(D)** SHIV-Q23AE and **(E)** SHIV-AD8-EO determined from the replication curves shown in (B) and (C), respectively. Data represent the average of four independent experiments, and error bars represent SEM. AUC values were compared using one-way analysis of variance (ANOVA) followed by Dunnett's multiple comparisons test. *** *p = 0*.*002*, * *p = 0*.*011*.

We have previously observed that the IFNα sensitivity of the SHIVs positively correlates with the replication capacity of the virus [25]. Thus, we hypothesized that basal levels of Ptm IFITMs have the potential to limit SHIV replication. We employed the *IFITM*-KO cell pools described above and assessed the ability of unadapted SHIV Q23AE and adapted SHIV AD8-EO to replicate in the absence of IFNα over a nine-day time course. Reduction of IFITM protein expression was confirmed through immunoblotting of KO cells. The levels of IFITM3/3A in the KO cells were lower than or comparable to the NTC cells (Fig 6A). Since the IFITM3/3A antibody does not distinguish between IFITM3 and IFITM3A, we are unable to rule out weather the observed IFITM levels in the *IFITM3* KO cells is due to the unedited cells in the KO pool or cross-detection of IFITM3A. Consistent with our previous results, we observed that the unadapted SHIV Q23AE replicates slowly with peak virus levels of ~10^4^ pg/ml of SIV p27 at six days post-infection (Fig 6B). In contrast, the adapted SHIV AD8-EO replicates rapidly reaching peak virus levels of >10^6^ pg/ml of SIV p27 by six days post-infection (Fig 6C). In order to compare the replication kinetics of the adapted and unadapted SHIVs across the *IFITM*-KO cells, we determined AUC for the replication curves from Fig 6B and 6C. When compared to the NTC cells (AUC 30.1 ± 0.6), a modest but statistically significant increase in SHIV Q23AE replication was observed in the *M1+M3* KO cells (AUC 32.1 ± 0.5, *p = 0*.*002*) and the *IFITM3* KO cells (31.6 ± 0.7, *p = 0*.*011*), despite comparable IFITM3/3A protein levels, but not in the *IFITM3A* KO cells (AUC 29.9 ± 0.6, Fig 6D). In contrast, *IFITM* KOs had no effect on the replication capacity of adapted SHIV AD8EO (Fig 6E). Thus, basal levels of IFITMs selectively limit replication of unadapted SHIV in macaque lymphocytes.

### Macaque IFITMs do not affect Env processing and incorporation in SHIV virions

We have observed that the unadapted SHIVs have 2.7- to 14.3-fold lower virion Env levels than adapted SHIVs and that virion Env content correlates with replication capacity and sensitivity to IFNα [25]. A recent study reported that the human IFITMs impair HIV-1 infectivity by decreasing Env processing in the host cell; increasing Env shedding; and reducing virion Env incorporation [41]. In order to determine the impact of Ptm IFITMs on Env processing, virion incorporation, and viral infectivity, we co-expressed SHIV AD8-EO or SHIV Q23AE and Ptm IFITM in HEK293T cells. The cell and virion lysate were immunoblotted and infectivity of cell-free virions were measured on TZM-bl reporter cell line. Consistent with other studies [37, 40, 43], we did not observe any differential effects of Ptm IFITM expression on Env processing in the producer cells and Env incorporation into viral particles (S4A and S4B Fig). In addition, Ptm IFITM expression did not affect infectivity of SHIV virions produced during 48 hours in these experiments (S4C Fig). This is perhaps not surprising given that the effect of IFN on replication is only apparent several days after infection in a spreading infection. Consistent with our previous study [25], we did observe that the unadapted SHIV Q23AE has lower virion Env levels than adapted SHIV AD8-EO and there are differences in the migration pattern of gp160/gp120, likely attributable to the glycosylation levels of the Env. Thus, our findings that the Ptm IFITMs limit replication of unadapted SHIV Q23AE during spreading infection lend support to the previously reported observations that IFITM-mediated restriction is most potent when IFITMs are present in both the donor and the target cells [30, 40].

## Discussion

Here we identify a novel example of cross-species restriction in which macaque-specific restriction factors selectively restrict replication of SHIV based on circulating, transmitted HIV-1 Env variant. In this study, we employed transcriptional profiling to define the repertoire of ISGs induced by IFNα in Ptm CD4+ lymphocytes. On the basis of this profiling, IFITMs, which have been implicated as anti-viral factors for a number of different viruses, including lentiviruses, were identified as a candidate restriction factors in these cells. We documented *IFITM* gene duplications in the macaque genomes and evaluated the ability of previously uncharacterized Ptm IFITMs to restrict SHIVs. We found that a prototypical, macaque-adapted SHIV is resistant to IFITM-mediated restriction, whereas a prototypical, unadapted SHIV, which encodes a circulating HIV-1 Env variant, is inhibited by Ptm IFITMs. Further, we demonstrate that IFITM virion incorporation tracks with the IFNα sensitivity of the SHIVs. For example, IFITMs are packaged at higher levels in IFNα-sensitive SHIVs when compared to IFNα-resistant SHIVs. Overall, our results suggest that the increased replication that results from adaptation of SHIVs in macaques may in part reflect an adaptation to an IFITM-mediated restriction.

The evolutionary “arms race” that results from antagonistic virus-host interactions has led to many evolutionary innovations that accelerate host adaptation to a virus [17]. The duplication of restriction factor genes is one such strategy through which the host is able to explore multiple evolutionary trajectories to select for advantageous mutations in restriction factor genes. For example, increase in the copy number of the restriction factor could allow the host to rapidly evolve against a number of different viruses and/or collectively target a given virus through different mechanisms to constrain viral evolution. Thus, it is not surprising that many restriction factor gene families, such as *APOBEC3* [44, 45], *Mx1* [46], and *TRIM5* [47, 48], have undergone gene duplications. In our RNA-seq dataset, we found sequence reads that uniquely map to multiple locations in the *IFITM* gene locus in the Ptm genome that are not found in the human genome, suggesting *IFITM* gene duplications. The duplication of IFITM genes has been previously reported for vertebrates [36] and a recent study reported recurrent duplication and divergence of IFITM3 in the primate genomes [37]. An in-depth analysis of IFITM gene duplications specific to the macaque genomes was lacking, though several of the macaque IFITM3 copies we describe were included in previous analyses (S5 Fig). Here, we demonstrate that macaques have three copies of *IFITM3* genes that map to three separate loci within the *IFITM* locus. We found that a duplicated *IFITM3* gene (LOC105494124, here called *IFITM3A*) encodes an ORF of the same length as the parental *IFITM3* gene (LOC105494127) that differs by 10.5% at the amino acid level. Moreover, *IFITM3A* is distinct from the human *IFITM3* duplication, named *IFITM2*, which is found in chimpanzees and gorillas in addition to humans [37]. Interestingly, we find that all three macaque species (pig-tailed, rhesus, and crab-eating) contain an *IFITM3* pseudogene within the *IFITM* locus, which is absent in humans. These pseudogene sequences group with high confidence in a clade that is separate from both the *IFITM1* and intact macaque *IFITM3*s (Fig 2B). Phylogenetically, we are unable to resolve whether these pseudogenes were born before or after the hominoid/Old World monkey split. Though, their presence in all three macaques and absence in humans suggests they were likely born in at least the last common ancestor of these macaques, but likely after the branching of Old World monkeys and hominoids. These pseudogene sequences are preceded by a long branch that presumably reflects a period of rapid but neutral evolution after birth or pseudogenization of this sequence. Together, these species restricted duplications of *IFITM3* (*IFITM2* in humans, chimps, gorillas, and *IFITM3A*, *IFITM3* pseudogene, and *IFITM3Ls* in macaques) suggest this gene may be recurrently duplicated during primate evolution. In addition to genes within the *IFITM* locus, our RNA-seq analysis identified expression of two *IFITM3*-like retrogenes (here called *IFITM3L*), which are present in the macaque genome on chromosome 16 and 9. These *IFITM3L* copies are both found in the intronic region of genes and have lost the intron present in *IFITM* sequences within the *IFITM* locus. These putative *IFITM* retrogenes await a described function.

In this study, we demonstrated that Ptm IFITMs are differentially incorporated in SHIV virions with higher levels in unadapted SHIVs in comparison to adapted SHIVs. It has been suggested that human IFITMs directly interact with HIV-1 Env [41], and their restriction activity maps to Env- and Gag-dependent virion incorporation [30]. Thus, it is plausible that the Envs encoded by unadapted SHIVs display greater colocalization and/or interaction with Ptm IFITMs. In contrast, the Envs encoded by adapted SHIVs evade and/or antagonize this interaction. Moreover, our data rule out the alternative possibility that the lower IFITM incorporation in the adapted SHIVs is due to reduced induction and/or degradation of IFITMs as similar steady-state and IFNα-induced levels of IFITM3 was observed in cells infected with adapted or unadapted SHIVs.

By generating *IFITM*-KO Ptm lymphocytes and infecting them with a prototype, macaque-adapted SHIV or a prototype, unadapted SHIV encoding HIV-1 Env isolated directly from an infected individual, we demonstrated that IFITMs partly contribute to the IFNα-induced inhibition of unadapted SHIV. We found that IFITM1 and parental IFITM3, but not the duplicated IFITM3A, determines IFNα sensitivity of unadapted SHIV. Because of the high sequence similarity between the Ptm *IFITMs*, the CRISPR guide RNA designed to target *IFITM1* also targeted *IFITM3/3A*. However, we could not discern the identity of *IFITM3s* (*IFITM3* and/or *IFITM3A)* that were targeted in these double “M1+M3” KO cells, as the only available anti-IFITM3 antibody does not distinguish between the two Ptm IFITM3s. Thus, due to the targeting of more than one *IFITM* by CRISPR guide RNA and because of the cross-reactivity of IFITM antibody, we cannot determine which specific IFITM contributes to anti-viral activity or whether multiple IFITMs are playing a role in inhibiting unadapted SHIV. Because there is extensive duplication of *IFITMs* in the macaque genome, including presence of *IFITM3L* genes on different chromosomes, we did not attempt combinatorial knockouts so as to avoid spurious effects due to gene-editing and recombination across multiple chromosomes. Our findings that *IFITM* KOs only result in partial recovery of viral replication of unadapted SHIVs to levels observed in the IFNα-untreated Ptm lymphocytes, suggest that either there is functional redundancy between Ptm IFITMs or ISGs other than IFITMs also contribute to IFNα-induced inhibition of unadapted SHIVs.

In a previous study, we found a significant positive correlation between viral replication kinetics and the sensitivity of SHIVs to IFNα in Ptm lymphocytes [25]. Here, we demonstrate that in contrast to adapted SHIV, depletion of basal levels of Ptm IFITMs increases the replication of unadapted SHIV in the absence of IFNα treatment. This suggests that the constitutively expressed IFITMs could contribute to the observed differences in the replication fitness of adapted vs unadapted SHIV, which could in turn affect their sensitivity to IFNα. Consistent with other reports [37, 40, 43]; we did not observe any differential effects of Ptm IFITM on Env processing and incorporation in SHIV virions. In contrast to the adapted SHIV, we did observe lower virion Env levels in the unadapted SHIV. Thus, one possibility is that lower virion Env levels in combination with IFITM virion packaging, decreases the replication kinetics of unadapted SHIVs rendering them sensitive to IFNα. In contrast, higher Env content and lower IFITM packaging in adapted SHIVs promotes higher replication, resulting in saturation of other IFNα-induced restriction factors. An alternative possibility is that high Env expression in cells infected with adapted SHIVs leads to increased cell-to-cell viral transmission thereby evading IFITM-mediated restriction. In support of this hypothesis, two recent studies demonstrated that in contrast to cell-free HIV-1 infection, cell-to-cell HIV-1 transmission renders it less sensitive to IFITM-mediated restriction [30, 49]. Thus, differences in the sensitivity of unadapted vs adapted SHIVs to Ptm IFITMs could also be explained by cell-to-cell transmission, raising the interesting possibility that the increased replication fitness of SHIVs adapted in macaques is a reflection of better cell-to-cell virus spread.

Adaptation of SHIVs to macaques typically involves serial macaque-passage to increase the replication capacity, transmissibility and pathogenicity [5–11]. As macaque IFNα-induced restriction factors can antagonize HIV-1 gene products encoded by SHIVs, successful adaptation of SHIVs in macaques likely involves overcoming these restrictions. This can potentially explain why adapted SHIVs cause persistent infection in macaques and the more biologically relevant unadapted SHIVs do not. If IFITMs constitute a selective force *in vivo* to control virus replication, then we hypothesize that the serial macaque-passage increases the replication capacity of SHIVs by overcoming IFITM-mediated restriction. Interestingly, such passaged viruses do become more resistant to IFNα inhibition with passage [25]. IFITMs may also play a role in restricting replication of simian tropic HIV-1 (stHIV-1) in macaque lymphocytes. In support of this hypothesis, two studies have suggested the presence of unidentified, IFNα-inducible host factor(s) that target stHIV-1 at an early stage of viral life cycle in Ptm lymphocytes [50, 51]. Moreover, these studies determined that the antiretroviral restriction factors—TRIM5, APOBEC3, BST-2/tetherin, and SAMHD1 are not responsible for this IFNα-induced restriction. Thus, it will be of interest to evaluate whether IFITMs restrict stHIV-1 in macaque lymphocytes. Lastly, it is well established that Envs from most transmitted HIV-1 strains demonstrate poor affinity for macaque CD4 [4] thus, SHIVs derived using such Envs replicate poorly, if at all, in macaque lymphocytes. Because it is difficult to study infection in unadapted SHIVs that lack mutations that have been identified to enhance macaque CD4 use [52, 53], we cannot draw conclusions on whether efficient engagement of macaque CD4 also affects IFITM-mediated restriction in macaques. Collectively, these studies may shed light on new approaches to further improve the SHIV/macaque models by rationally designing SHIVs to avoid key macaque restriction factors while maintaining as much as possible of the HIV-1 character of the virus.

## Materials and methods

### Cells, viruses, transfections

HEK293T (ATCC CRL-3216), IFITMdel Mouse Embryonic Fibroblasts (MEFs) [38], and HeLa TZM-bl cells (NIH AIDS Reagent program catalog no. 8129) were cultured in Dulbecco's modified eagle medium (DMEM, Gibco) supplemented with 10% fetal bovine serum (FBS, Gibco), 2 mM L-glutamine (Gibco), and 1x Anti-anti (anti-microbial/anti-mycotic, Gibco). Immortalized pig-tailed macaque (Ptm) CD4+ lymphocytes [54] were cultured in Iscove’s modified Dulbecco’s medium (IMDM) supplemented with 10% FBS, 2 mM L-glutamine, 1x Anti-anti, and 100 U/ml of interleukin-2 (Roche) (complete IMDM).

The following full-length proviral plasmids were used to generate viruses used in this study: SHIV AD8-EO, SHIV AD8-OG, SHIV 1157ipd3N4, SHIV QF495AE, SHIV Q23AE, SHIV MG505GV and SHIV BG505AE (S3 Table). Replication-competent SHIVs were generated by transfecting 2x10^6^ HEK293T cells with 4 μg of proviral plasmid DNA and 12 μl of Fugene 6 transfection reagent (Roche) following manufacturer's protocol. Forty-eight hours post-transfection, virus-containing supernatant was harvested, passed through a 0.2 μm sterile filter and concentrated ~10-fold using Amicon Ultracel 100 kDa filters (Millipore). Replication-competent stock of SHIV SF162P3 [6] was generated by expanding the virus in immortalized Ptm lymphocytes as described previously [25]. Aliquots of replication-competent SHIV stocks were stored at -80°C. The viral titer of each SHIV stock was determined by infecting TZM-bl cells and staining for β-galactosidase activity 48 hours post-infection [55].

For transient co-transfection experiments, HEK293T cells (2.5x10^6^ cells/well in a 6-well plate) were seeded 24 hours prior to transfection. Cells were co-transfected with plasmids encoding SHIV proviral DNA (1 μg) and Ptm FLAG-IFITM variant or an empty vector control (0.5 μg) using Fugene 6 transfection reagent (Roche) following manufacturer's protocol. Six replicate wells were transfected per sample. Forty-eight hours post-transfection, cells and virus-containing supernatant were harvested for subsequent analysis.

### RNA-seq of Ptm lymphocytes

RNA was extracted and purified from the Ptm lymphocytes using the RNeasy Mini kit (Qiagen) following manufacturer's protocol. Total RNA integrity was checked using an Agilent 2200 TapeStation (Agilent Technologies) and quantified using a Trinean DropSense96 spectrophotometer (Caliper Life Sciences). RNA-seq libraries were prepared from total RNA using the TruSeq RNA Sample Prep Kit v2 (Illumina) and a Sciclone NGSx Workstation (PerkinElmer). Library size distributions were validated using an Agilent 2200 TapeStation (Agilent Technologies). Additional library QC, blending of pooled indexed libraries, and cluster optimization were performed using Life Technologies’ Invitrogen Qubit 2.0 Fluorometer (Life Technologies-Invitrogen). RNA-seq libraries were pooled (6-plex) onto a flow cell lane. Sequencing was performed using an Illumina HiSeq 2500 in rapid mode employing a paired-end, 50 base read length (PE50) sequencing strategy. Image analysis and base calling were performed using Illumina's Real Time Analysis v1.18 software, followed by 'demultiplexing' of indexed reads and generation of FASTQ files, using Illumina's bcl2fastq Conversion Software v1.8.4.

### RNA-seq data analysis

Reads of low quality were filtered prior to alignment to Mnem 1.0 using TopHat v2.1.0 [56]. Counts were generated from TopHat alignments for each gene using the Python package HTSeq v0.6.1p1 [57]. Genes with low counts across all samples were removed, prior to identification of differentially expressed genes using the Bioconductor package edgeR v3.12.0 [58]. A false discovery rate (FDR) method was employed to correct for multiple testing [59], with differential expression defined as |log_2_ (ratio) | ≥ 0.585 (± 1.5-fold) with the FDR set to 5%. To predict which proteins may contain transmembrane helices, TMHMM v2.0 [35] was used with amino acid sequences from genes identified as being significantly differentially expressed. All RNA-sequencing FTP data files are available from the NCBI GEO database (accession number GSE126594).

### IFITM phylogenetic analysis

Human IFITM1, IFITM2, IFITM3, IFITM5 genes were collected from NCBI (NM_003641, NM_006435, NM_021034, NM_001025295). The IFITM locus for each macaque species was mapped using BLAT on UCSC genome browser (for *M*. *mulatta*/BCM Mmul_8.0.1/rheMac8 and *M*. *fascicularis*/Macaca_fascicularis_5.0/macFas5) or BLASTN (for *M*. *nemestrina*/Mnem_1.0) and extracting the aligned sequences. An alignment of IFITM nucleotide sequences from the three macaque species and humans was created using MAFFT with auto algorithm parameters [60] within Geneious version 11.1.4 [61]. The IFITM phylogenetic tree was created using PHYML with NNIs topology search, BioNJ initial tree, HKY85 nucleotide substitution model, and 100 bootstraps [62]. Synteny maps were built using pairwise dotplots (in Geneious) of the IFITM locus amongst species in addition to the pattern of sequence grouping in the phylogenetic tree.

### Immunoblotting

Whole cell extracts were prepared by lysing the cells in radioimmunoprecipitation assay (RIPA) cell lysis buffer (50 mM Tris pH 8.0, 0.1% SDS, 1% Triton-X, 150 mM NaCl, 1% deoxycholic acid, 2 mM PMSF). For virion incorporation, virus containing supernatants from the infected or transfected cell cultures were centrifuged at 650 x g for five minutes at room temperature. Cell-free supernatant was filtered through 0.2 μm filter and then pelleted through a 25% sucrose cushion by ultracentrifugation for at 28,000 rpm for 90 minutes at 4°C. Virus pellets were lysed in 70 μl of RIPA buffer for 10 minutes at room temperature. The concentration of SIV p27 in the viral lysates was determined by SIV p27 antigen ELISA (Advanced BioScience Laboratories), and normalized amounts of lysate were subjected to SDS-PAGE and immunoblotted. Standard Western blotting procedures were used with the following antibodies: SIV p27 (ABL catalog no. 4323), IFITM3 (Proteintech catalog no. 11714-1-AP), IFITM1 (Proteintech catalog no. 11727-3-AP), GAPDH (BioRad catalog no. MCA4739P), FLAG (OriGene catalog no. TA100023), and HIV-1 gp120 (NIH AIDS Reagent program catalog no. 288). The IFITM3 antibody (Proteintech catalog no. 11714-1-AP) used in this study reacts with both Ptm IFITM3 and Ptm IFITM3A (S2 Fig); therefore, the immunoblots using this antibody are labeled as “IFITM3/3A”. Protein expression was quantified by measuring the band intensities using ImageJ software.

### Purification of viral particles using sucrose density gradient fractionation

Sucrose density gradient fractionation was performed as described previously [63] with some modifications. Briefly, 8x10^6^ Ptm lymphocytes were infected at an MOI of 0.02 by spinoculation at 1200 x g for 90 minutes at room temperature. After spinoculation, cells were washed 4x with 1 ml of complete IMDM, re-suspended in 9.2 ml of complete IMDM and plated in two wells of a 6-well plate. Five hours after the initial infection, IFNα-2a (PBL Assay Science) was added to the culture at a final concentration of 1,000 U/ml. Every three days, two-third of the culture was replaced with fresh, complete IMDM containing IFNα-2a. On day 9 post-infection, the virus containing supernatant from infected cultures were collected, filtered through 0.2 μm filter and pelleted through a 25% sucrose cushion by ultracentrifugation at 28,000 rpm for 2 hours at 4°C. Pelleted virions were resuspended in 200 μl phosphate-buffered saline (PBS), loaded on 20–70% (w/v) linear sucrose density gradients in PBS, and ultracentrifuged at 35,000 rpm for 16 hours at 4°C in SW41 rotor (Beckman). Ten 1 ml fractions were collected from the top of the gradient, equilibrated in 10% trichloroacetic acid, and subjected to SDS-PAGE and immunoblotted with SIV p27 and IFITM3 antibodies.

### Generation of IFITM knockout cell pools

IFITM knockout cell pools were generated by electroporation of CRISPR/Cas9 ribonucleoproteins (crRNPs) as described previously [64] with some modification. Briefly, IFITM knockout cell pools were generated by electroporating Ptm lymphocytes with custom IFITM targeting crRNAs (Table 1). crRNA (IDT) and tracrRNA (IDT catalog no. 1072534) were resuspended at 160 μM in 10 mM Tris pH 7.4. 1 μl crRNA was complexed at an equimolar ratio with 1 μl tracrRNA and incubated for 30 minutes at 37°C followed by addition of 2 μl of 40 μM Cas9-NLS (UC Berkeley MacroLab) and further incubation at 37°C for 15 minutes to create the IFITM-targeting crRNP complexes. 3.5 μl crRNP was added to 5x10^5^ Ptm lymphocytes resuspended in Amaxa SG Cell Line 96-well Nucleofector Kit (Lonza catalog no V4SC-3096) and electroporated using a Lonza 4D Nucleofector according to the manufacturer’s protocol. After electroporation, 80 μl of prewarmed complete IMDM was added, followed by 30-minute recovery by incubating at 37°C. Eight replicate electroporations were carried out for each crRNA. Following recovery, cells from the eight replicate electroporations were pooled and resuspended at a density of 1.6x10^6^ cells/mL in 2.5 ml complete IMDM in a 12-well plate. IFITM knockout was analyzed by Western blotting at 3, 7 and 14 days post-electroporation.

### SHIV replication time course

Replication of SHIVs was accessed as described previously [25]. Briefly, 1x10^6^ Ptm lymphocytes were infected at an MOI of 0.02 by spinoculation at 1200 x g for 90 minutes at room temperature. After spinoculation, cells were washed 4x with 1 ml of complete IMDM, re-suspended in 1.2 ml of complete IMDM and plated in two wells of a 48-well plate. Five hours after the initial infection, IFNα-2a (PBL Assay Science) was added to one well at a final concentration of 1,000 U/ml. Every three days, two-third of the cultures were harvested and cell-free supernatant were separated by pelleting at 650 x g for five minutes at room temperature. Cultures were replenished with fresh, complete IMDM, including with IFNα-2a if appropriate. SIV p27 concentrations were determined using a SIV p27 antigen ELISA (Advanced BioScience Laboratories). The data and statistical analyses were performed using Prism version 6.0c (GraphPad Software).

### Immunofluorescent Microscopy

For imaging experiments, 10^5^ HEK293T cells or IFITMdel MEFs were grown on sterilized glass coverslips in 12-well plates and transfected with 500 ng of plasmid encoding Ptm HA-IFITM variant or an empty vector control using Fugene 6 transfection reagent (Roche) following manufacturer's protocol. 24 hours post-transfection, cells were fixed with 4% paraformaldehyde in phosphate buffered saline (PBS) for 20 minutes, permeabilized with 0.1% Triton X100 in PBS for 10 minutes, and blocked with 2% FBS in PBS for 10 minutes. Cells were stained with the anti-HA primary antibody (1:1000, HA.11 Covance) and Alexa Fluor 488-conjugated goat anti-mouse secondary antibody (1:1000, Life Technologies). Coverslips were mounted with Prolong Gold Antifade reagent containing DAPI (Life Technologies) and images were taken using an Olympus Fluoview FV10i confocal microscope.

## Supporting information

S1 Fig Mapping of RNA-seq reads to the macaque *IFITMs*. Representative coverage plots for the three *IFITM* gene loci in the IFNα-treated and -untreated samples. The y axis indicates the number of reads and the x axis indicates the Ptm genomic location (NW_012011633 Unplaced Scaffold Reference Mnem_1.0). The *IFITM* gene locus is indicated on the top left of each sample set. The total read coverage, which includes all reads (unambiguous and ambiguous) aligned to the region, is shown in grey. The coverage computed using only uniquely mapping reads is overlaid in red.
(PDF)

S2 Fig Expression and detection of exogenous macaque IFITMs using anti-IFITM3/3A antibody. Western blot analysis of Ptm FLAG-IFITM expression in HEK293T cells using anti-FLAG (*left panel*) and anti-IFITM3/3A (*right panel*) antibodies. Cells transfected with plasmid that express the indicated Ptm FLAG-IFITM or an empty vector control (indicated “EV”) is labeled at the top.
(PDF)

S3 Fig Localization of macaque IFITMs. Immunofluorescence images of Ptm HA-IFITM localization in Mouse Embryonic Fibroblasts lacking the IFITM locus (IFITMdel MEFs) and HEK293T cells stained with anti-HA antibody (in green) and DAPI (blue). Cells transfected with plasmid that express the indicated Ptm HA-IFITM or an empty vector control (indicated “EV”) is labeled on the left.
(PDF)

S4 Fig Macaque IFITMs do not affect Env processing and incorporation into viral particles. **(A)** Western blot analysis of HEK293T cells co-transfected with plasmids that express the indicated SHIV and the indicated Ptm FLAG-IFITM or an empty vector control (indicated “EV”). **(B)** Western blot analysis of cell-free SHIV virions from (A). Virions equivalent to 20 ng of SIV p27 was loaded into each lane. Immunoblotting performed using anti-FLAG, anti-HIV-1 gp120 288, anti-SIV Gag p27, and anti-GAPDH antibodies. **(C)** Infectivity of cell-free SHIV virions equivalent to 100 pg of SIV p27 from (A) measured on the TZM-bl reporter assay. The y-axis represents the relative β-galactosidase units and the x-axis represents the indicated SHIV.
(PDF)

S5 Fig Alignment of macaque and human IFITMs. A nucleotide alignment of open reading frame (ORF)-containing IFITMs from Fig 2 and all IFITM3-annotated sequences from a previously described study by Compton *et al*. [37]. Translation of each nucleotide sequences and a maximum likelihood tree of the sequences are also shown.
(PDF)

S1 File The nucleotide alignment and maximum likelihood tree for IFITM1, IFITM3, and IFITM3-related sequences from macaques and humans. (NEX)

S1 Table List of significantly differentially expressed genes. (PDF)

S2 Table List of upregulated genes encoding transmembrane helices. (PDF)

S3 Table SHIVs used in this study. (PDF)
