# Supplementary figures and images for: Macaque interferon-induced transmembrane proteins limit replication of SHIV strains in an Envelope-dependent manner
Source: PLoS Pathog. 2019 Jul 1;15(7):e1007925. doi: 10.1371/journal.ppat.1007925 (PMC6625738; doi:10.1371/journal.ppat.1007925)

### IFITM3A (LOC105494124)

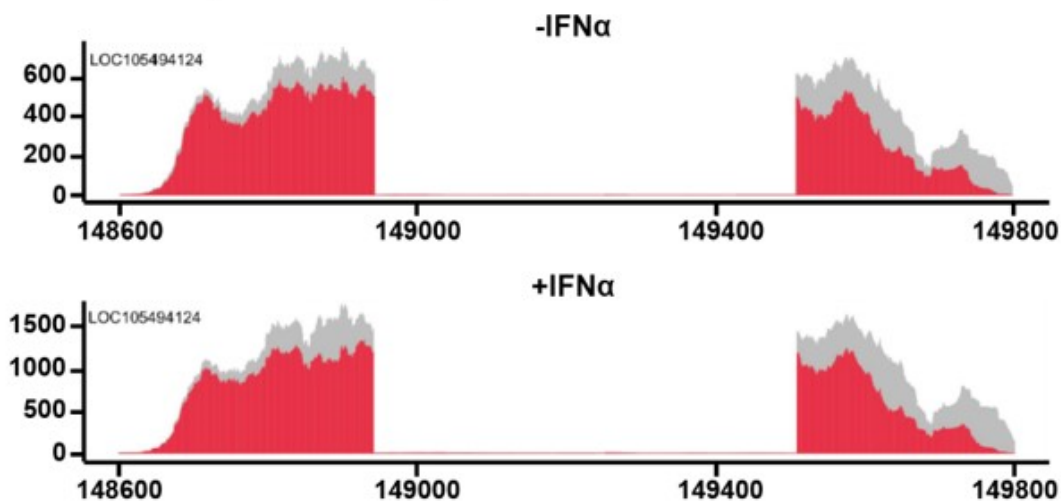

### IFITM3 (LOC105494127)

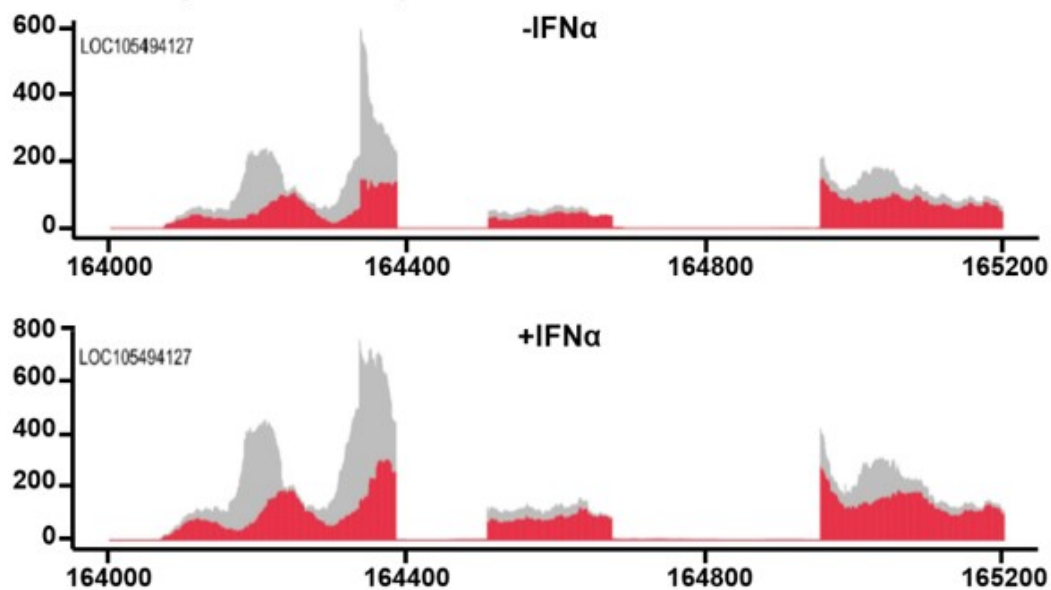

### IFITM1

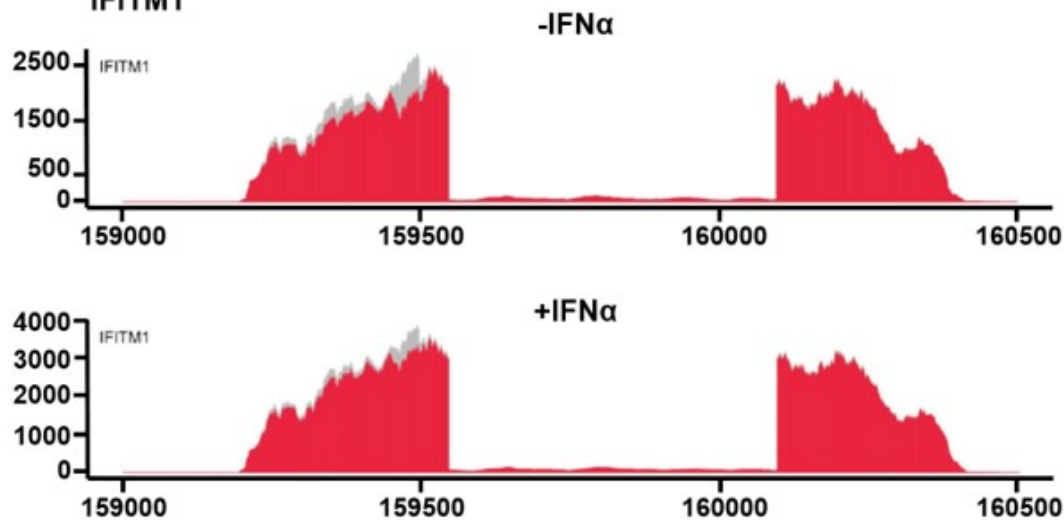

Supplement: S1 Fig — Representative coverage plots for the three IFITM gene loci in the IFNα-treated and -untreated samples. The y axis indicates the number of reads and the x axis indicates the Ptm genomic location (NW_012011633 Unplaced Scaffold Reference Mnem_1.0). The IFITM gene locus is indicated on the top left of each sample set. The total read coverage, which includes all reads (unambiguous and ambiguous) aligned to the region, is shown in grey. The coverage computed using only uniquely mapping reads is overlaid in red. (PDF) [file ppat.1007925.s001.pdf]

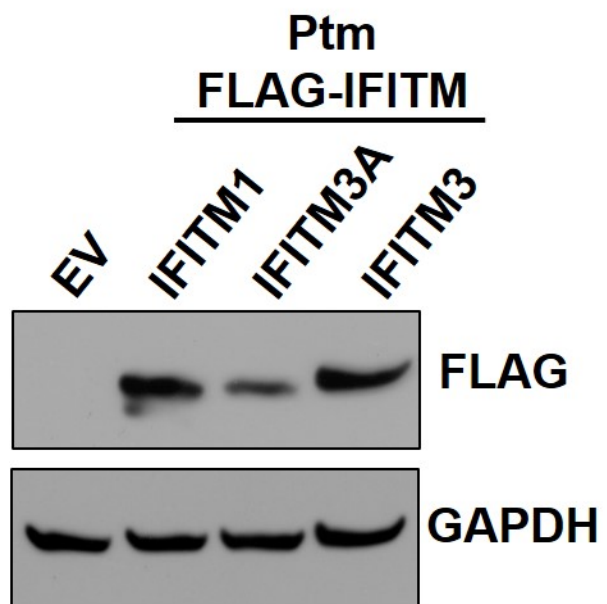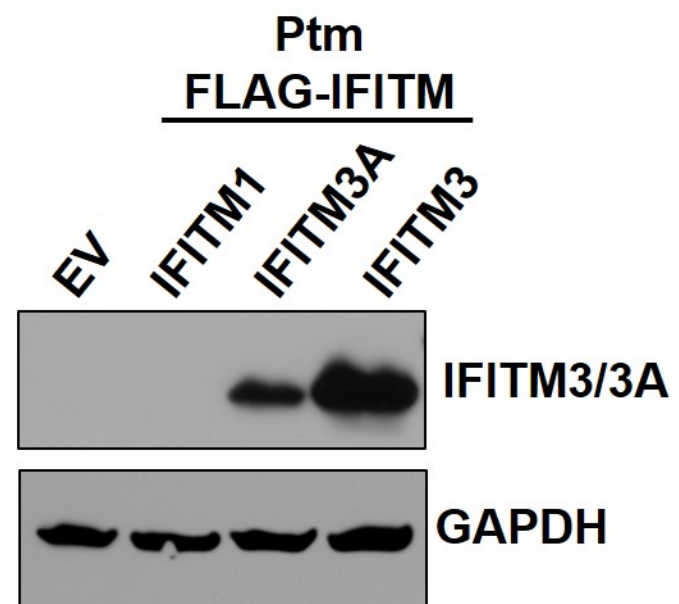

Supplement: S2 Fig — Western blot analysis of Ptm FLAG-IFITM expression in HEK293T cells using anti-FLAG (left panel) and anti-IFITM3/3A (right panel) antibodies. Cells transfected with plasmid that express the indicated Ptm FLAG-IFITM or an empty vector control (indicated “EV”) is labeled at the top. (PDF) [file ppat.1007925.s002.pdf]

IFITMdel MEFs

HEK293T

EV

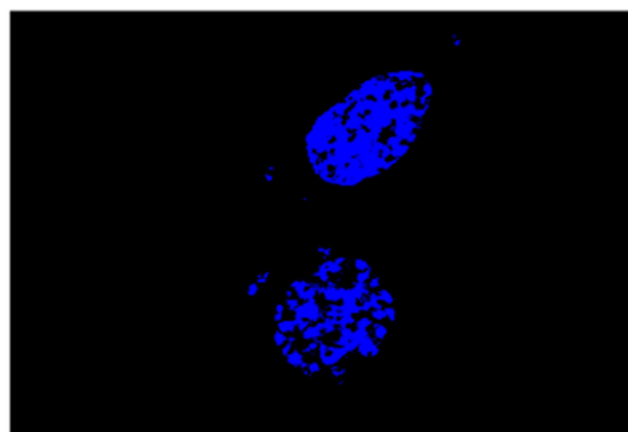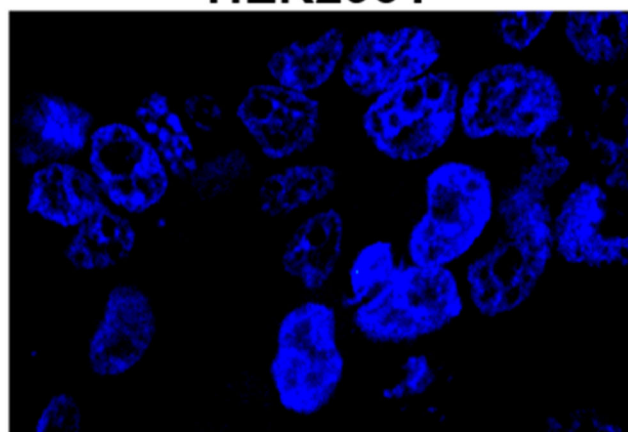

HA-IFITM1

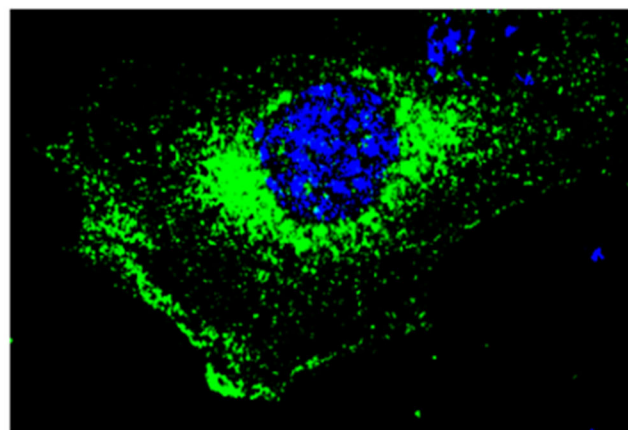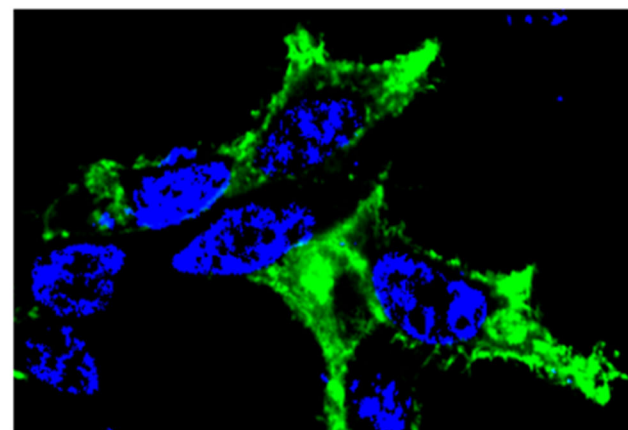

HA-IFITM3

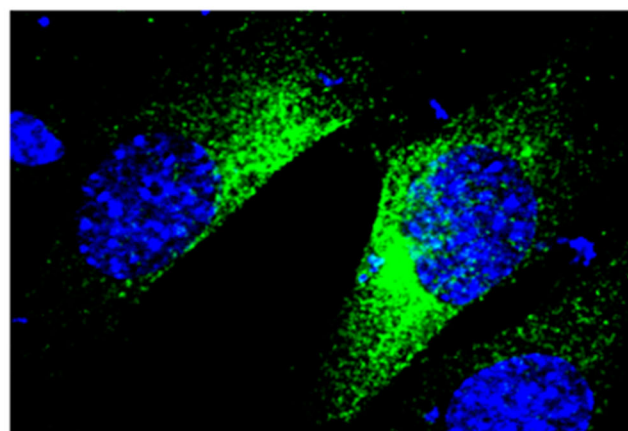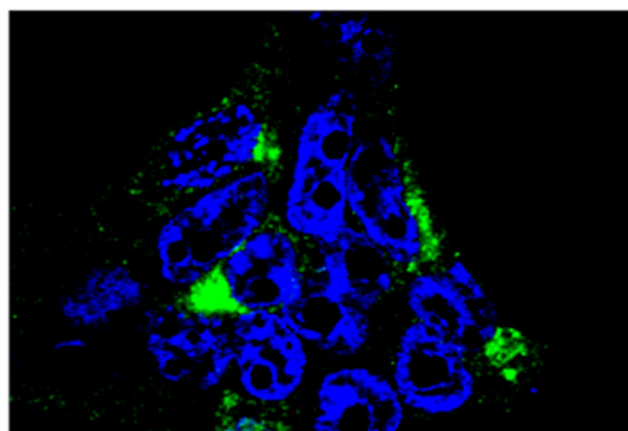

HA-IFITM3A

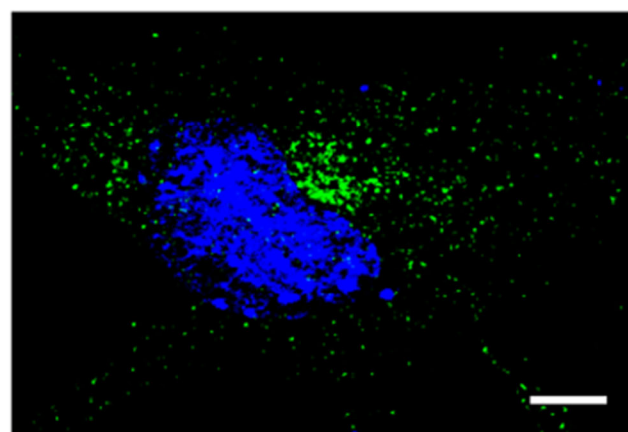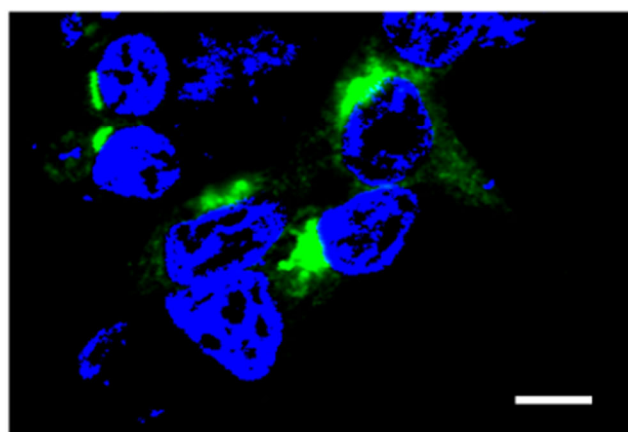

Supplement: S3 Fig — Immunofluorescence images of Ptm HA-IFITM localization in Mouse Embryonic Fibroblasts lacking the IFITM locus (IFITMdel MEFs) and HEK293T cells stained with anti-HA antibody (in green) and DAPI (blue). Cells transfected with plasmid that express the indicated Ptm HA-IFITM or an empty vector control (indicated “EV”) is labeled on the left. (PDF) [file ppat.1007925.s003.pdf]

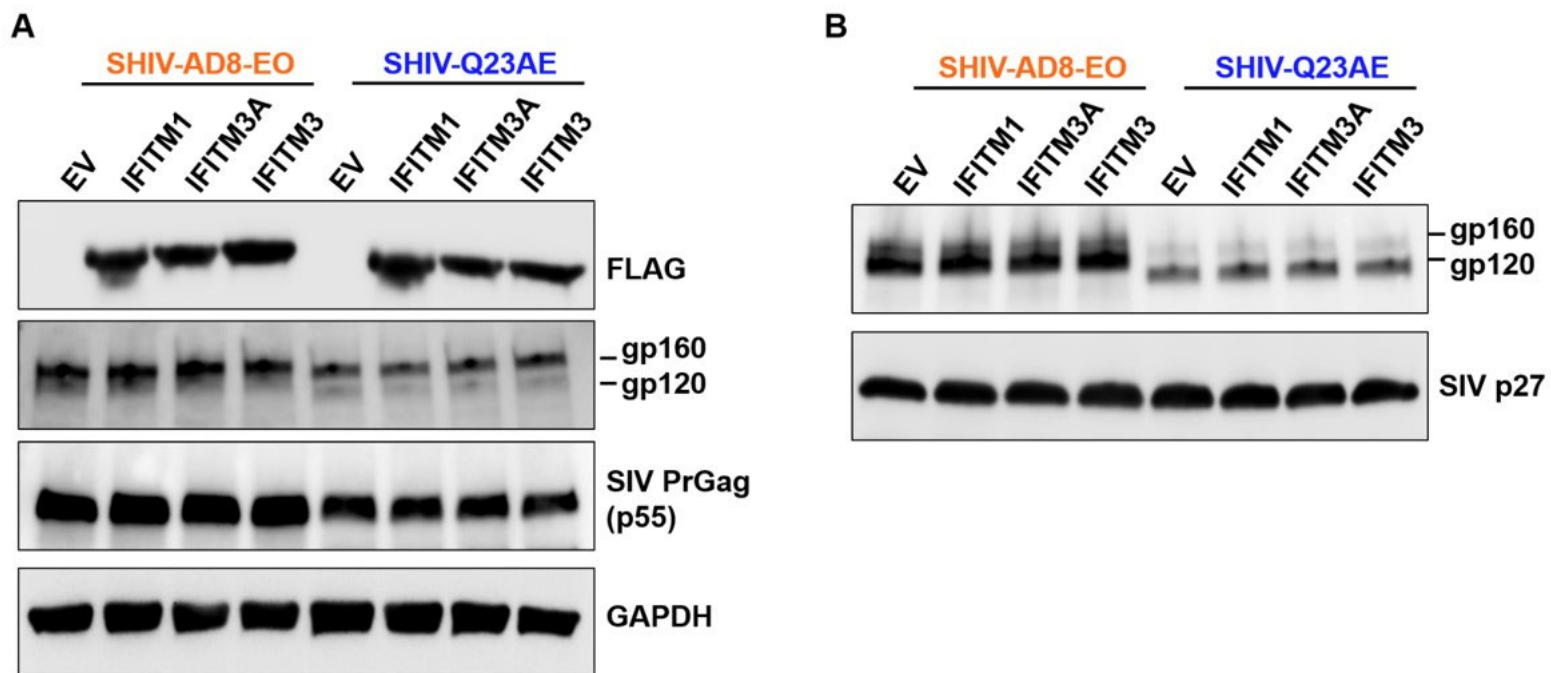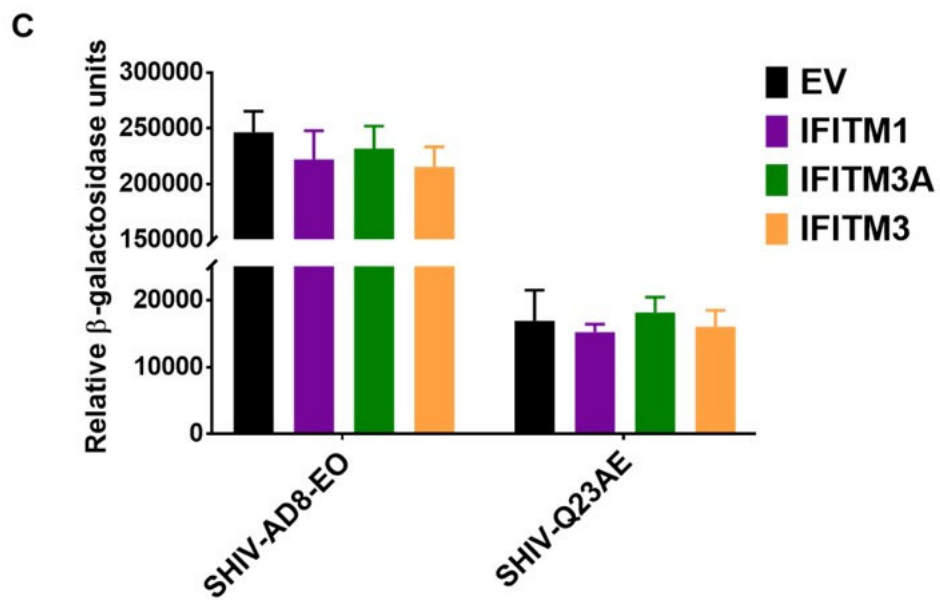

Supplement: S4 Fig — (A) Western blot analysis of HEK293T cells co-transfected with plasmids that express the indicated SHIV and the indicated Ptm FLAG-IFITM or an empty vector control (indicated “EV”). (B) Western blot analysis of cell-free SHIV virions from (A). Virions equivalent to 20 ng of SIV p27 was loaded into each lane. Immunoblotting performed using anti-FLAG, anti-HIV-1 gp120 288, anti-SIV Gag p27, and anti-GAPDH antibodies. (C) Infectivity of cell-free SHIV virions equivalent to 100 pg of SIV p27 from (A) measured on the TZM-bl reporter assay. The y-axis represents the relative β-galactosidase units and the x-axis represents the indicated SHIV. (PDF) [file ppat.1007925.s004.pdf]
