# Supplementary material for: Macaque interferon-induced transmembrane proteins limit replication of SHIV strains in an Envelope-dependent manner
Source: PLoS Pathog. 2019 Jul 1;15(7):e1007925. doi: 10.1371/journal.ppat.1007925 (PMC6625738; doi:10.1371/journal.ppat.1007925)
Supplement: S5 Fig — A nucleotide alignment of open reading frame (ORF)-containing IFITMs from Fig 2 and all IFITM3-annotated sequences from a previously described study by Compton et al. [37]. Translation of each nucleotide sequences and a maximum likelihood tree of the sequences are also shown. (PDF) [file ppat.1007925.s005.pdf]

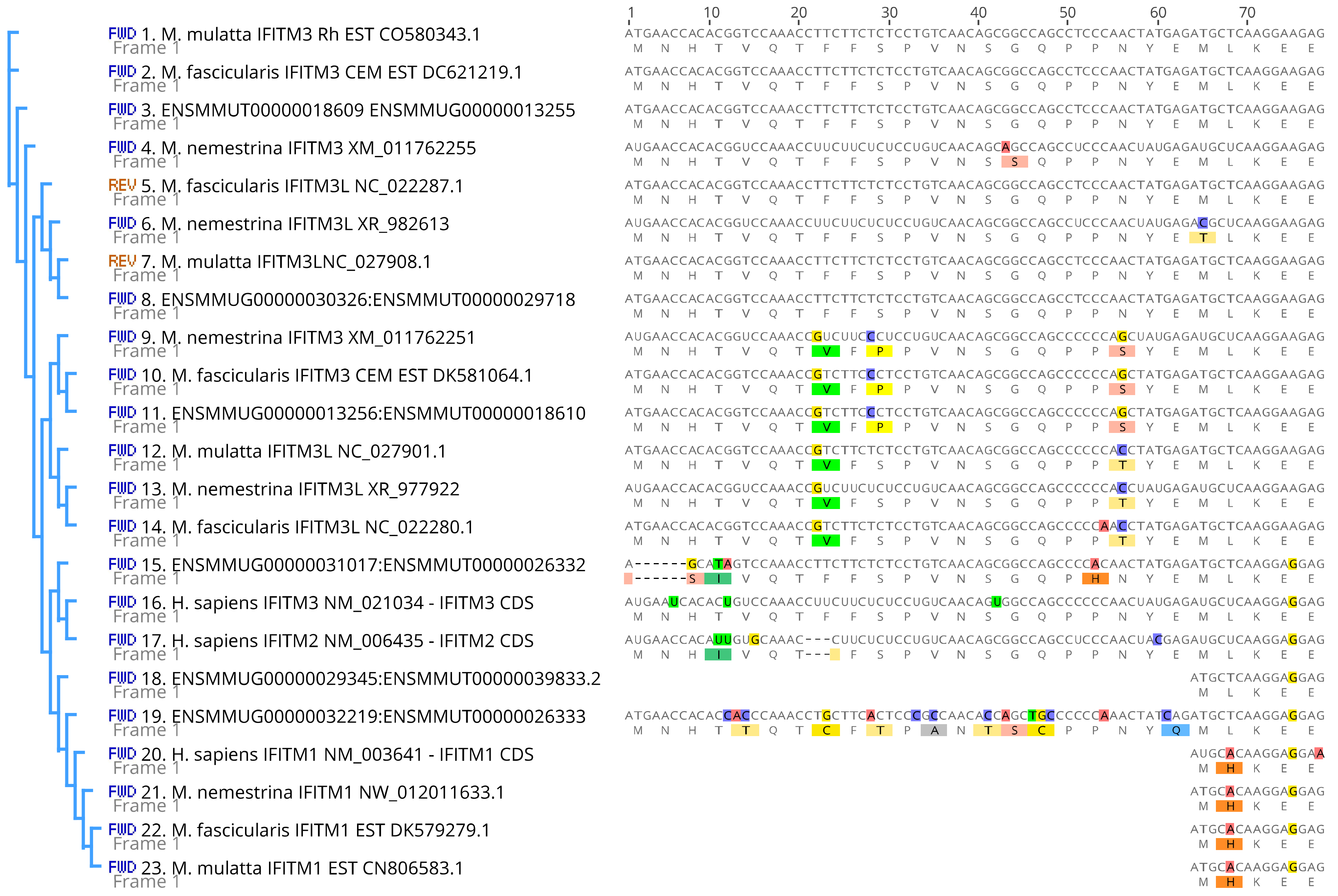
1 10 20 30 40 50 60 70
FWD 1. M. mulatta IFITM3 Rh EST CO580343.1
Frame 1
ATGAACCACACGGTCCAAACCTTCTTCTCTCCTGTCAACAGCGGCCAGCCTCCCAACTATGAGATGCTCAAGGAAGAG
M N H T V Q T F F S P V N S G Q P P N Y E M L K E E
FWD 2. M. fascicularis IFITM3 CEM EST DC621219.1
Frame 1
ATGAACCACACGGTCCAAACCTTCTTCTCTCCTGTCAACAGCGGCCAGCCTCCCAACTATGAGATGCTCAAGGAAGAG
M N H T V Q T F F S P V N S G Q P P N Y E M L K E E
FWD 3. ENSMMUT00000018609 ENSMMUG00000013255
Frame 1
ATGAACCACACGGTCCAAACCTTCTTCTCTCCTGTCAACAGCGGCCAGCCTCCCAACTATGAGATGCTCAAGGAAGAG
M N H T V Q T F F S P V N S G Q P P N Y E M L K E E
FWD 4. M. nemestrina IFITM3 XM_011762255
Frame 1
AUGAACCACACGGUCCAAACCUUCUUCUCUCCUGUCAACAGCAGCCAGCCUCCCAACUAUGAGAUGCUCAAGGAAGAG
M N H T V Q T F F S P V N S S Q P P N Y E M L K E E
REV 5. M. fascicularis IFITM3L NC_022287.1
Frame 1
ATGAACCACACGGTCCAAACCTTCTTCTCTCCTGTCAACAGCGGCCAGCCTCCCAACTATGAGATGCTCAAGGAAGAG
M N H T V Q T F F S P V N S G Q P P N Y E M L K E E
FWD 6. M. nemestrina IFITM3L XR_982613
Frame 1
AUGAACCACACGGUCCAAACCUUCUUCUCUCCUGUCAACAGCGGCCAGCCUCCCAACUAUGAGACGCUCAAGGAAGAG
M N H T V Q T F F S P V N S G Q P P N Y E T L K E E
REV 7. M. mulatta IFITM3LNC_027908.1
Frame 1
ATGAACCACACGGTCCAAACCTTCTTCTCTCCTGTCAACAGCGGCCAGCCTCCCAACTATGAGATGCTCAAGGAAGAG
M N H T V Q T F F S P V N S G Q P P N Y E M L K E E
FWD 8. ENSMMUG00000030326:ENSMMUT00000029718
Frame 1
ATGAACCACACGGTCCAAACCTTCTTCTCTCCTGTCAACAGCGGCCAGCCTCCCAACTATGAGATGCTCAAGGAAGAG
M N H T V Q T F F S P V N S G Q P P N Y E M L K E E
FWD 9. M. nemestrina IFITM3 XM_011762251
Frame 1
AUGAACCACACGGUCCAAACCGUCUUCCCUCCUGUCAACAGCGGCCAGCCCCCCAGCUAUGAGAUGCUCAAGGAAGAG
M N H T V Q T V F P P V N S G Q P P S Y E M L K E E
FWD 10. M. fascicularis IFITM3 CEM EST DK581064.1
Frame 1
ATGAACCACACGGTCCAAACCGTCTTCCCTCCTGTCAACAGCGGCCAGCCCCCCAGCTATGAGATGCTCAAGGAAGAG
M N H T V Q T V F P P V N S G Q P P S Y E M L K E E
FWD 11. ENSMMUG00000013256:ENSMMUT00000018610
Frame 1
ATGAACCACACGGTCCAAACCGTCTTCCCTCCTGTCAACAGCGGCCAGCCCCCCAGCTATGAGATGCTCAAGGAAGAG
M N H T V Q T V F P P V N S G Q P P S Y E M L K E E
FWD 12. M. mulatta IFITM3L NC_027901.1
Frame 1
ATGAACCACACGGTCCAAACCGTCTTCTCTCCTGTCAACAGCGGCCAGCCCCCCACCTATGAGATGCTCAAGGAAGAG
M N H T V Q T V F S P V N S G Q P P T Y E M L K E E
FWD 13. M. nemestrina IFITM3L XR_977922
Frame 1
AUGAACCACACGGUCCAAACCGUCUUCUCUCCUGUCAACAGCGGCCAGCCCCCCACCUAUGAGAUGCUCAAGGAAGAG
M N H T V Q T V F S P V N S G Q P P T Y E M L K E E
FWD 14. M. fascicularis IFITM3L NC_022280.1
Frame 1
ATGAACCACACGGTCCAAACCGTCTTCTCTCCTGTCAACAGCGGCCAGCCCCCAACCTATGAGATGCTCAAGGAAGAG
M N H T V Q T V F S P V N S G Q P P T Y E M L K E E
FWD 15. ENSMMUG00000031017:ENSMMUT00000026332
Frame 1
A------GCATAGTCCAAACCTTCTTCTCTCCTGTCAACAGCGGCCAGCCCCACAACTATGAGATGCTCAAGGAGGAG
------S I V Q T F F S P V N S G Q P H N Y E M L K E E
FWD 16. H. sapiens IFITM3 NM_021034 - IFITM3 CDS
Frame 1
AUGAAUCACACUGUCCAAACCUUCUUCUCUCCUGUCAACAGUGGCCAGCCCCCCAACUAUGAGAUGCUCAAGGAGGAG
M N H T V Q T F F S P V N S G Q P P N Y E M L K E E
FWD 17. H. sapiens IFITM2 NM_006435 - IFITM2 CDS
Frame 1
AUGAACCACAUUGUGCAAAC---CUUCUCUCCUGUCAACAGCGGCCAGCCUCCCAACUACGAGAUGCUCAAGGAGGAG
M N H I V Q T--- F S P V N S G Q P P N Y E M L K E E
FWD 18. ENSMMUG00000029345:ENSMMUT00000039833.2
Frame 1
ATGCTCAAGGAGGAG
M L K E E
FWD 19. ENSMMUG00000032219:ENSMMUT00000026333
Frame 1
ATGAACCACACCACCCAAACCTGCTTCACTCCCGCCAACACCAGCTGCCCCCCAAACTATCAGATGCTCAAGGAGGAG
M N H T T Q T C F T P A N T S C P P N Y Q M L K E E
FWD 20. H. sapiens IFITM1 NM_003641 - IFITM1 CDS
Frame 1
AUGCACAAGGAGGAA
M H K E E
FWD 21. M. nemestrina IFITM1 NW_012011633.1
Frame 1
ATGCACAAGGAGGAG
M H K E E
FWD 22. M. fascicularis IFITM1 EST DK579279.1
Frame 1
ATGCACAAGGAGGAG
M H K E E
FWD 23. M. mulatta IFITM1 EST CN806583.1
Frame 1
ATGCACAAGGAGGAG
M H K E E

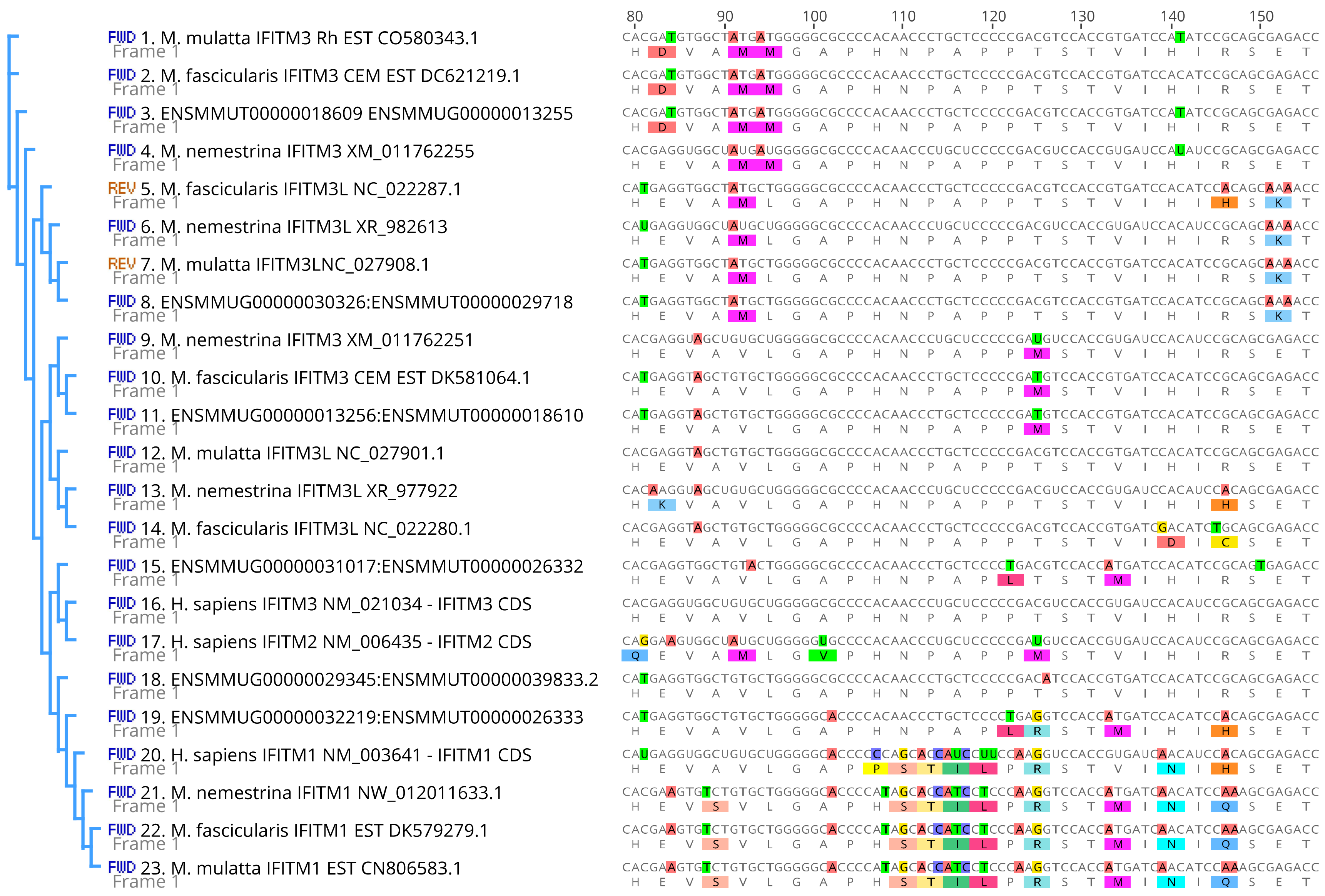
80
90
100
110
120
130
140
150
FWD 1. M. mulatta IFITM3 Rh EST CO580343.1
Frame 1
FWD 2. M. fascicularis IFITM3 CEM EST DC621219.1
Frame 1
FWD 3. ENSMMUT00000018609 ENSMMUG00000013255
Frame 1
FWD 4. M. nemestrina IFITM3 XM_011762255
Frame 1
REV 5. M. fascicularis IFITM3L NC_022287.1
Frame 1
FWD 6. M. nemestrina IFITM3L XR_982613
Frame 1
REV 7. M. mulatta IFITM3LNC_027908.1
Frame 1
FWD 8. ENSMMUG00000030326:ENSMMUT00000029718
Frame 1
FWD 9. M. nemestrina IFITM3 XM_011762251
Frame 1
FWD 10. M. fascicularis IFITM3 CEM EST DK581064.1
Frame 1
FWD 11. ENSMMUG00000013256:ENSMMUT00000018610
Frame 1
FWD 12. M. mulatta IFITM3L NC_027901.1
Frame 1
FWD 13. M. nemestrina IFITM3L XR_977922
Frame 1
FWD 14. M. fascicularis IFITM3L NC_022280.1
Frame 1
FWD 15. ENSMMUG00000031017:ENSMMUT00000026332
Frame 1
FWD 16. H. sapiens IFITM3 NM_021034 - IFITM3 CDS
Frame 1
FWD 17. H. sapiens IFITM2 NM_006435 - IFITM2 CDS
Frame 1
FWD 18. ENSMMUG00000029345:ENSMMUT00000039833.2
Frame 1
FWD 19. ENSMMUG00000032219:ENSMMUT00000026333
Frame 1
FWD 20. H. sapiens IFITM1 NM_003641 - IFITM1 CDS
Frame 1
FWD 21. M. nemestrina IFITM1 NW_012011633.1
Frame 1
FWD 22. M. fascicularis IFITM1 EST DK579279.1
Frame 1
FWD 23. M. mulatta IFITM1 EST CN806583.1
Frame 1

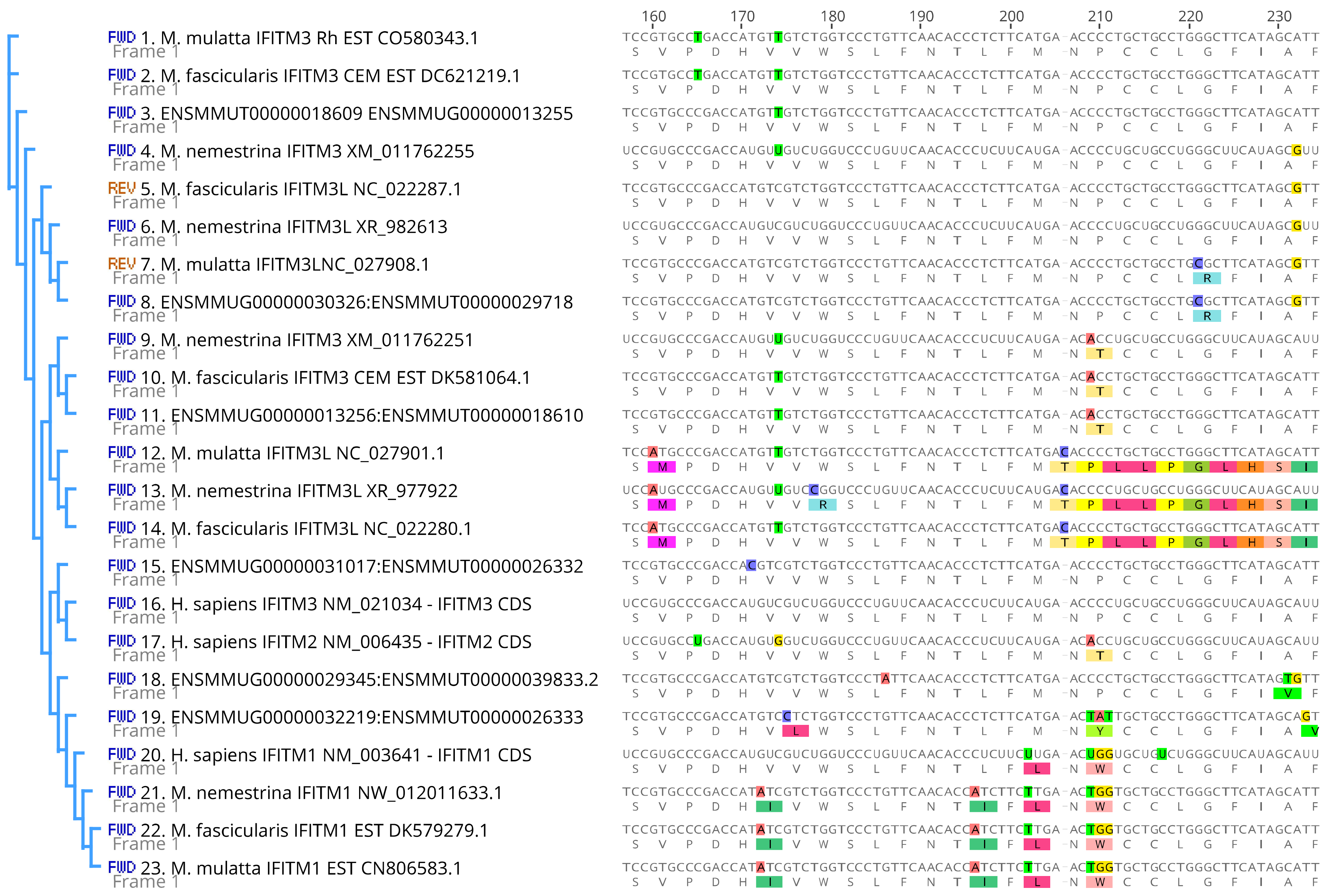

160 170 180 190 200 210 220 230
FWD 1. M. mulatta IFITM3 Rh EST CO580343.1
Frame 1
TCCGTGCCTGACCATGTTGTCTGGTCCCTGTTCAACACCCTCTTCATGA-ACCCCTGCTGCCTGGGCTTCATAGCATT
S V P D H V V W S L F N T L F M -N P C C L G F I A F
FWD 2. M. fascicularis IFITM3 CEM EST DC621219.1
Frame 1
TCCGTGCCTGACCATGTTGTCTGGTCCCTGTTCAACACCCTCTTCATGA-ACCCCTGCTGCCTGGGCTTCATAGCATT
S V P D H V V W S L F N T L F M -N P C C L G F I A F
FWD 3. ENSMMUT00000018609 ENSMMUG00000013255
Frame 1
TCCGTGCCCGACCATGTTGTCTGGTCCCTGTTCAACACCCTCTTCATGA-ACCCCTGCTGCCTGGGCTTCATAGCATT
S V P D H V V W S L F N T L F M -N P C C L G F I A F
FWD 4. M. nemestrina IFITM3 XM_011762255
Frame 1
UCCGUGCCCGACCAUGUUGUCUGGUCCCUGUUCAACACCCUCUUCAUGA-ACCCCUGCUGCCUGGGCUUCAUAGCGUU
S V P D H V V W S L F N T L F M -N P C C L G F I A F
REV 5. M. fascicularis IFITM3L NC_022287.1
Frame 1
TCCGTGCCCGACCATGTCGTCTGGTCCCTGTTCAACACCCTCTTCATGA-ACCCCTGCTGCCTGGGCTTCATAGCGTT
S V P D H V V W S L F N T L F M -N P C C L G F I A F
FWD 6. M. nemestrina IFITM3L XR_982613
Frame 1
UCCGUGCCCGACCAUGUCGUCUGGUCCCUGUUCAACACCCUCUUCAUGA-ACCCCUGCUGCCUGGGCUUCAUAGCGUU
S V P D H V V W S L F N T L F M -N P C C L G F I A F
REV 7. M. mulatta IFITM3LNC_027908.1
Frame 1
TCCGTGCCCGACCATGTCGTCTGGTCCCTGTTCAACACCCTCTTCATGA-ACCCCTGCTGCCTGCGCTTCATAGCGTT
S V P D H V V W S L F N T L F M -N P C C L R F I A F
FWD 8. ENSMMUG00000030326:ENSMMUT00000029718
Frame 1
TCCGTGCCCGACCATGTCGTCTGGTCCCTGTTCAACACCCTCTTCATGA-ACCCCTGCTGCCTGCGCTTCATAGCGTT
S V P D H V V W S L F N T L F M -N P C C L R F I A F
FWD 9. M. nemestrina IFITM3 XM_011762251
Frame 1
UCCGUGCCCGACCAUGUUGUCUGGUCCCUGUUCAACACCCUCUUCAUGA-ACACCUGCUGCCUGGGCUUCAUAGCAUU
S V P D H V V W S L F N T L F M -N T C C L G F I A F
FWD 10. M. fascicularis IFITM3 CEM EST DK581064.1
Frame 1
TCCGTGCCCGACCATGTTGTCTGGTCCCTGTTCAACACCCTCTTCATGA-ACACCTGCTGCCTGGGCTTCATAGCATT
S V P D H V V W S L F N T L F M -N T C C L G F I A F
FWD 11. ENSMMUG00000013256:ENSMMUT00000018610
Frame 1
TCCGTGCCCGACCATGTTGTCTGGTCCCTGTTCAACACCCTCTTCATGA-ACACCTGCTGCCTGGGCTTCATAGCATT
S V P D H V V W S L F N T L F M -N T C C L G F I A F
FWD 12. M. mulatta IFITM3L NC_027901.1
Frame 1
TCCATGCCCGACCATGTTGTCTGGTCCCTGTTCAACACCCTCTTCATGACACCCCTGCTGCCTGGGCTTCATAGCATT
S M P D H V V W S L F N T L F M T P L L P G L H S I
FWD 13. M. nemestrina IFITM3L XR_977922
Frame 1
UCCAUGCCCGACCAUGUUGUCCGGUCCCUGUUCAACACCCUCUUCAUGACACCCCUGCUGCCUGGGCUUCAUAGCAUU
S M P D H V V R S L F N T L F M T P L L P G L H S I
FWD 14. M. fascicularis IFITM3L NC_022280.1
Frame 1
TCCATGCCCGACCATGTTGTCTGGTCCCTGTTCAACACCCTCTTCATGACACCCCTGCTGCCTGGGCTTCATAGCATT
S M P D H V V W S L F N T L F M T P L L P G L H S I
FWD 15. ENSMMUG00000031017:ENSMMUT00000026332
Frame 1
TCCGTGCCCGACCACGTCGTCTGGTCCCTGTTCAACACCCTCTTCATGA-ACCCCTGCTGCCTGGGCTTCATAGCATT
S V P D H V V W S L F N T L F M -N P C C L G F I A F
FWD 16. H. sapiens IFITM3 NM_021034 - IFITM3 CDS
Frame 1
UCCGUGCCCGACCAUGUCGUCUGGUCCCUGUUCAACACCCUCUUCAUGA-ACCCCUGCUGCCUGGGCUUCAUAGCAUU
S V P D H V V W S L F N T L F M -N P C C L G F I A F
FWD 17. H. sapiens IFITM2 NM_006435 - IFITM2 CDS
Frame 1
UCCGUGCCUGACCAUGUGGUCUGGUCCCUGUUCAACACCCUCUUCAUGA-ACACCUGCUGCCUGGGCUUCAUAGCAUU
S V P D H V V W S L F N T L F M -N T C C L G F I A F
FWD 18. ENSMMUG00000029345:ENSMMUT00000039833.2
Frame 1
TCCGTGCCCGACCATGTCGTCTGGTCCCTATTCAACACCCTCTTCATGA-ACCCCTGCTGCCTGGGCTTCATAGTGTT
S V P D H V V W S L F N T L F M -N P C C L G F I V F
FWD 19. ENSMMUG00000032219:ENSMMUT00000026333
Frame 1
TCCGTGCCCGACCATGTCCTCTGGTCCCTGTTCAACACCCTCTTCATGA-ACTATTGCTGCCTGGGCTTCATAGCAGT
S V P D H V L W S L F N T L F M -N Y C C L G F I A V
FWD 20. H. sapiens IFITM1 NM_003641 - IFITM1 CDS
Frame 1
UCCGUGCCCGACCAUGUCGUCUGGUCCCUGUUCAACACCCUCUUCUUGA-ACUGGUGCUGUCUGGGCUUCAUAGCAUU
S V P D H V V W S L F N T L F L -N W C C L G F I A F
FWD 21. M. nemestrina IFITM1 NW_012011633.1
Frame 1
TCCGTGCCCGACCATATCGTCTGGTCCCTGTTCAACACCATCTTCTTGA-ACTGGTGCTGCCTGGGCTTCATAGCATT
S V P D H I V W S L F N T I F L -N W C C L G F I A F
FWD 22. M. fascicularis IFITM1 EST DK579279.1
Frame 1
TCCGTGCCCGACCATATCGTCTGGTCCCTGTTCAACACCATCTTCTTGA-ACTGGTGCTGCCTGGGCTTCATAGCATT
S V P D H I V W S L F N T I F L -N W C C L G F I A F
FWD 23. M. mulatta IFITM1 EST CN806583.1
Frame 1
TCCGTGCCCGACCATATCGTCTGGTCCCTGTTCAACACCATCTTCTTGA-ACTGGTGCTGCCTGGGCTTCATAGCATT
S V P D H I V W S L F N T I F L -N W C C L G F I A F

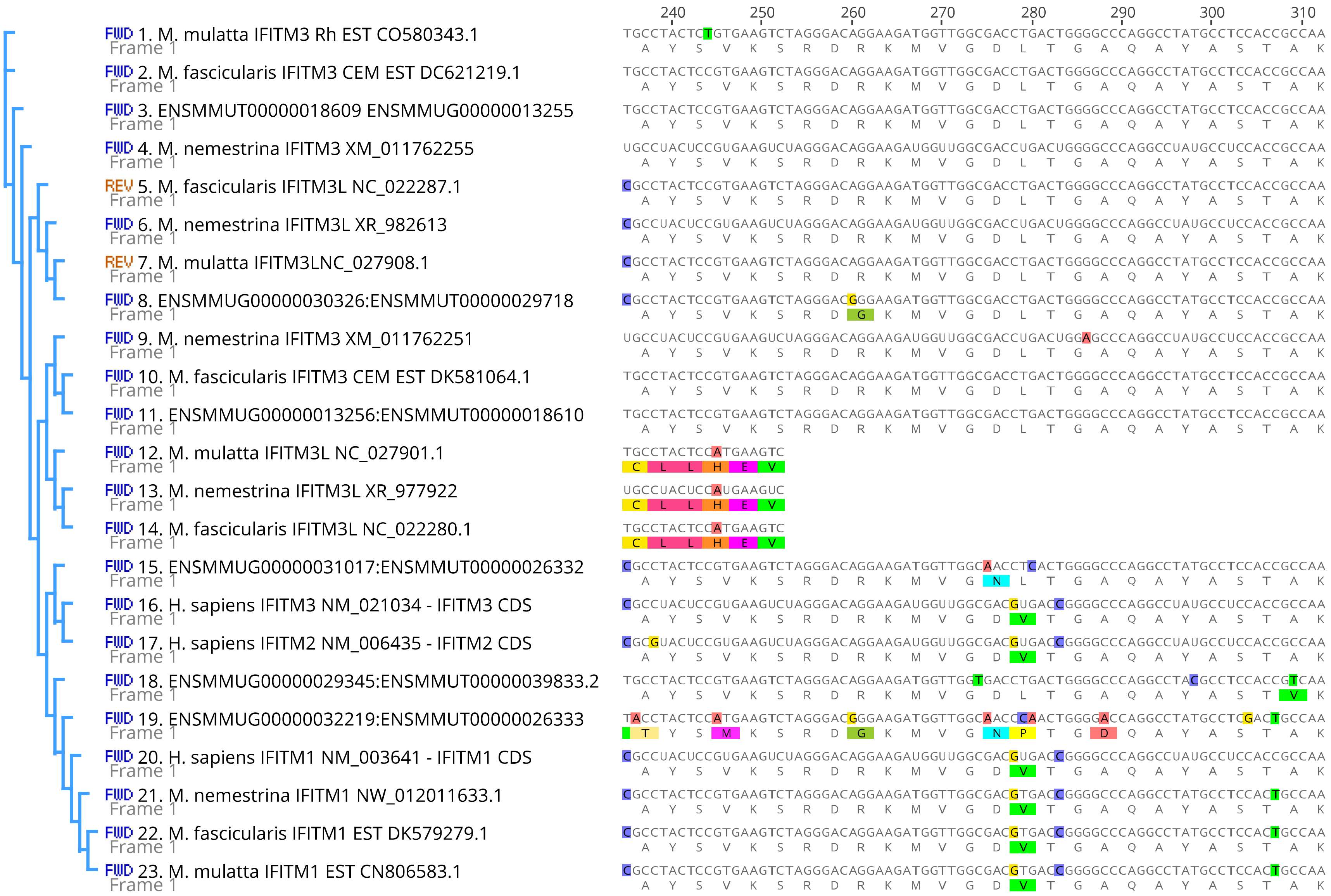
240 250 260 270 280 290 300 310
FWD 1. M. mulatta IFITM3 Rh EST CO580343.1
Frame 1
TGCCTACTCTGTGAAGTCTAGGGACAGGAAGATGGTTGGCGACCTGACTGGGGCCCAGGCCTATGCCTCCACCGCCAA
A Y S V K S R D R K M V G D L T G A Q A Y A S T A K
FWD 2. M. fascicularis IFITM3 CEM EST DC621219.1
Frame 1
TGCCTACTCCGTGAAGTCTAGGGACAGGAAGATGGTTGGCGACCTGACTGGGGCCCAGGCCTATGCCTCCACCGCCAA
A Y S V K S R D R K M V G D L T G A Q A Y A S T A K
FWD 3. ENSMMUT00000018609 ENSMMUG00000013255
Frame 1
TGCCTACTCCGTGAAGTCTAGGGACAGGAAGATGGTTGGCGACCTGACTGGGGCCCAGGCCTATGCCTCCACCGCCAA
A Y S V K S R D R K M V G D L T G A Q A Y A S T A K
FWD 4. M. nemestrina IFITM3 XM_011762255
Frame 1
UGCCUACUCCGUGAAGUCUAGGGACAGGAAGAUGGUUGGCGACCUGACUGGGGCCCAGGCCUAUGCCUCCACCGCCAA
A Y S V K S R D R K M V G D L T G A Q A Y A S T A K
REV 5. M. fascicularis IFITM3L NC_022287.1
Frame 1
CGCCTACTCCGTGAAGTCTAGGGACAGGAAGATGGTTGGCGACCTGACTGGGGCCCAGGCCTATGCCTCCACCGCCAA
A Y S V K S R D R K M V G D L T G A Q A Y A S T A K
FWD 6. M. nemestrina IFITM3L XR_982613
Frame 1
CGCCUACUCCGUGAAGUCUAGGGACAGGAAGAUGGUUGGCGACCUGACUGGGGCCCAGGCCUAUGCCUCCACCGCCAA
A Y S V K S R D R K M V G D L T G A Q A Y A S T A K
REV 7. M. mulatta IFITM3LNC_027908.1
Frame 1
CGCCTACTCCGTGAAGTCTAGGGACAGGAAGATGGTTGGCGACCTGACTGGGGCCCAGGCCTATGCCTCCACCGCCAA
A Y S V K S R D R K M V G D L T G A Q A Y A S T A K
FWD 8. ENSMMUG00000030326:ENSMMUT00000029718
Frame 1
CGCCTACTCCGTGAAGTCTAGGGACGGGAAGATGGTTGGCGACCTGACTGGGGCCCAGGCCTATGCCTCCACCGCCAA
A Y S V K S R D G K M V G D L T G A Q A Y A S T A K
FWD 9. M. nemestrina IFITM3 XM_011762251
Frame 1
UGCCUACUCCGUGAAGUCUAGGGACAGGAAGAUGGUUGGCGACCUGACUGGAGCCCAGGCCUAUGCCUCCACCGCCAA
A Y S V K S R D R K M V G D L T G A Q A Y A S T A K
FWD 10. M. fascicularis IFITM3 CEM EST DK581064.1
Frame 1
TGCCTACTCCGTGAAGTCTAGGGACAGGAAGATGGTTGGCGACCTGACTGGGGCCCAGGCCTATGCCTCCACCGCCAA
A Y S V K S R D R K M V G D L T G A Q A Y A S T A K
FWD 11. ENSMMUG00000013256:ENSMMUT00000018610
Frame 1
TGCCTACTCCGTGAAGTCTAGGGACAGGAAGATGGTTGGCGACCTGACTGGGGCCCAGGCCTATGCCTCCACCGCCAA
A Y S V K S R D R K M V G D L T G A Q A Y A S T A K
FWD 12. M. mulatta IFITM3L NC_027901.1
Frame 1
TGCCTACTCCATGAAGTC
C L L H E V
FWD 13. M. nemestrina IFITM3L XR_977922
Frame 1
UGCCUACUCCAUGAAGUC
C L L H E V
FWD 14. M. fascicularis IFITM3L NC_022280.1
Frame 1
TGCCTACTCCATGAAGTC
C L L H E V
FWD 15. ENSMMUG00000031017:ENSMMUT00000026332
Frame 1
CGCCTACTCCGTGAAGTCTAGGGACAGGAAGATGGTTGGCAACCTCACTGGGGCCCAGGCCTATGCCTCCACCGCCAA
A Y S V K S R D R K M V G N L T G A Q A Y A S T A K
FWD 16. H. sapiens IFITM3 NM_021034 - IFITM3 CDS
Frame 1
CGCCUACUCCGUGAAGUCUAGGGACAGGAAGAUGGUUGGCGACGUGACCGGGGCCCAGGCCUAUGCCUCCACCGCCAA
A Y S V K S R D R K M V G D V T G A Q A Y A S T A K
FWD 17. H. sapiens IFITM2 NM_006435 - IFITM2 CDS
Frame 1
CGCGUACUCCGUGAAGUCUAGGGACAGGAAGAUGGUUGGCGACGUGACCGGGGCCCAGGCCUAUGCCUCCACCGCCAA
A Y S V K S R D R K M V G D V T G A Q A Y A S T A K
FWD 18. ENSMMUG00000029345:ENSMMUT00000039833.2
Frame 1
TGCCTACTCCGTGAAGTCTAGGGACAGGAAGATGGTTGGTGACCTGACTGGGGCCCAGGCCTACGCCTCCACCGTCAA
A Y S V K S R D R K M V G D L T G A Q A Y A S T V K
FWD 19. ENSMMUG00000032219:ENSMMUT00000026333
Frame 1
TACCTACTCCATGAAGTCTAGGGACGGGAAGATGGTTGGCAACCCAACTGGGGACCAGGCCTATGCCTCGACTGCCAA
T Y S M K S R D G K M V G N P T G D Q A Y A S T A K
FWD 20. H. sapiens IFITM1 NM_003641 - IFITM1 CDS
Frame 1
CGCCUACUCCGUGAAGUCUAGGGACAGGAAGAUGGUUGGCGACGUGACCGGGGCCCAGGCCUAUGCCUCCACCGCCAA
A Y S V K S R D R K M V G D V T G A Q A Y A S T A K
FWD 21. M. nemestrina IFITM1 NW_012011633.1
Frame 1
CGCCTACTCCGTGAAGTCTAGGGACAGGAAGATGGTTGGCGACGTGACCGGGGCCCAGGCCTATGCCTCCACTGCCAA
A Y S V K S R D R K M V G D V T G A Q A Y A S T A K
FWD 22. M. fascicularis IFITM1 EST DK579279.1
Frame 1
CGCCTACTCCGTGAAGTCTAGGGACAGGAAGATGGTTGGCGACGTGACCGGGGCCCAGGCCTATGCCTCCACTGCCAA
A Y S V K S R D R K M V G D V T G A Q A Y A S T A K
FWD 23. M. mulatta IFITM1 EST CN806583.1
Frame 1
CGCCTACTCCGTGAAGTCTAGGGACAGGAAGATGGTTGGCGACGTGACCGGGGCCCAGGCCTATGCCTCCACTGCCAA
A Y S V K S R D R K M V G D V T G A Q A Y A S T A K

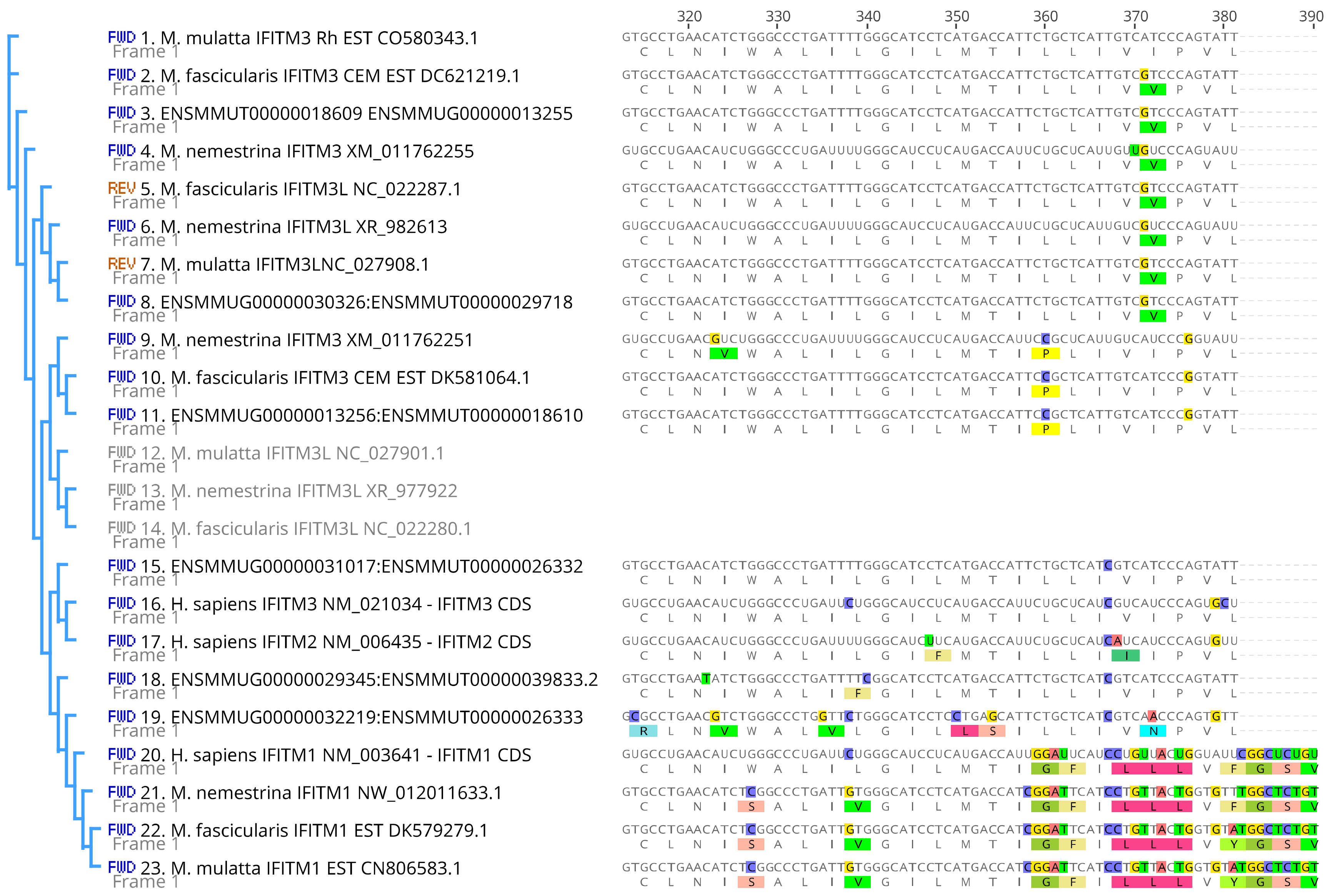

320 330 340 350 360 370 380 390
FWD 1. M. mulatta IFITM3 Rh EST CO580343.1
Frame 1
GTGCCTGAACATCTGGGCCCTGATTTTGGGCATCCTCATGACCATTCTGCTCATTGTCATCCCAGTATT
C L N I W A L I L G I L M T I L L I V I P V L
FWD 2. M. fascicularis IFITM3 CEM EST DC621219.1
Frame 1
GTGCCTGAACATCTGGGCCCTGATTTTGGGCATCCTCATGACCATTCTGCTCATTGTCGTCCCAGTATT
C L N I W A L I L G I L M T I L L I V V P V L
FWD 3. ENSMMUT00000018609 ENSMMUG00000013255
Frame 1
GTGCCTGAACATCTGGGCCCTGATTTTGGGCATCCTCATGACCATTCTGCTCATTGTCGTCCCAGTATT
C L N I W A L I L G I L M T I L L I V V P V L
FWD 4. M. nemestrina IFITM3 XM_011762255
Frame 1
GUGCCUGAACAUCUGGGCCCUGAUUUUGGGCAUCCUCAUGACCAUUCUGCUCAUUGUUGUCCCAGUAUU
C L N I W A L I L G I L M T I L L I V V P V L
REV 5. M. fascicularis IFITM3L NC_022287.1
Frame 1
GTGCCTGAACATCTGGGCCCTGATTTTGGGCATCCTCATGACCATTCTGCTCATTGTCGTCCCAGTATT
C L N I W A L I L G I L M T I L L I V V P V L
FWD 6. M. nemestrina IFITM3L XR_982613
Frame 1
GUGCCUGAACAUCUGGGCCCUGAUUUUGGGCAUCCUCAUGACCAUUCUGCUCAUUGUCGUCCCAGUAUU
C L N I W A L I L G I L M T I L L I V V P V L
REV 7. M. mulatta IFITM3LNC_027908.1
Frame 1
GTGCCTGAACATCTGGGCCCTGATTTTGGGCATCCTCATGACCATTCTGCTCATTGTCGTCCCAGTATT
C L N I W A L I L G I L M T I L L I V V P V L
FWD 8. ENSMMUG00000030326:ENSMMUT00000029718
Frame 1
GTGCCTGAACATCTGGGCCCTGATTTTGGGCATCCTCATGACCATTCTGCTCATTGTCGTCCCAGTATT
C L N I W A L I L G I L M T I L L I V V P V L
FWD 9. M. nemestrina IFITM3 XM_011762251
Frame 1
GUGCCUGAACGUCUGGGCCCUGAUUUUGGGCAUCCUCAUGACCAUUCCGCUCAUUGUCAUCCCGGUAUU
C L N V W A L I L G I L M T I P L I V I P V L
FWD 10. M. fascicularis IFITM3 CEM EST DK581064.1
Frame 1
GTGCCTGAACATCTGGGCCCTGATTTTGGGCATCCTCATGACCATTCCGCTCATTGTCATCCCGGTATT
C L N I W A L I L G I L M T I P L I V I P V L
FWD 11. ENSMMUG00000013256:ENSMMUT00000018610
Frame 1
GTGCCTGAACATCTGGGCCCTGATTTTGGGCATCCTCATGACCATTCCGCTCATTGTCATCCCGGTATT
C L N I W A L I L G I L M T I P L I V I P V L
FWD 12. M. mulatta IFITM3L NC_027901.1
Frame 1
FWD 13. M. nemestrina IFITM3L XR_977922
Frame 1
FWD 14. M. fascicularis IFITM3L NC_022280.1
Frame 1
FWD 15. ENSMMUG00000031017:ENSMMUT00000026332
Frame 1
GTGCCTGAACATCTGGGCCCTGATTTTGGGCATCCTCATGACCATTCTGCTCATCGTCATCCCAGTATT
C L N I W A L I L G I L M T I L L I V I P V L
FWD 16. H. sapiens IFITM3 NM_021034 - IFITM3 CDS
Frame 1
GUGCCUGAACAUCUGGGCCCUGAUUCUGGGCAUCCUCAUGACCAUUCUGCUCAUCGUCAUCCCAGUGCU
C L N I W A L I L G I L M T I L L I V I P V L
FWD 17. H. sapiens IFITM2 NM_006435 - IFITM2 CDS
Frame 1
GUGCCUGAACAUCUGGGCCCUGAUUUUGGGCAUCUUCAUGACCAUUCUGCUCAUCAUCAUCCCAGUGUU
C L N I W A L I L G I F M T I L L I I I P V L
FWD 18. ENSMMUG00000029345:ENSMMUT00000039833.2
Frame 1
GTGCCTGAATATCTGGGCCCTGATTTTCGGCATCCTCATGACCATTCTGCTCATCGTCATCCCAGTATT
C L N I W A L I F G I L M T I L L I V I P V L
FWD 19. ENSMMUG00000032219:ENSMMUT00000026333
Frame 1
GCGCCTGAACGTCTGGGCCCTGGTTCTGGGCATCCTCCTGAGCATTCTGCTCATCGTCAACCCAGTGTT
R L N V W A L V L G I L L S I L L I V N P V L
FWD 20. H. sapiens IFITM1 NM_003641 - IFITM1 CDS
Frame 1
GUGCCUGAACAUCUGGGCCCUGAUUCUGGGCAUCCUCAUGACCAUUGGAUUCAUCCUGUUACUGGUAUUCGGCUCUGU
C L N I W A L I L G I L M T I G F I L L L V F G S V
FWD 21. M. nemestrina IFITM1 NW_012011633.1
Frame 1
GTGCCTGAACATCTCGGCCCTGATTGTGGGCATCCTCATGACCATCGGATTCATCCTGTTACTGGTGTTTGGCTCTGT
C L N I S A L I V G I L M T I G F I L L L V F G S V
FWD 22. M. fascicularis IFITM1 EST DK579279.1
Frame 1
GTGCCTGAACATCTCGGCCCTGATTGTGGGCATCCTCATGACCATCGGATTCATCCTGTTACTGGTGTATGGCTCTGT
C L N I S A L I V G I L M T I G F I L L L V Y G S V
FWD 23. M. mulatta IFITM1 EST CN806583.1
Frame 1
GTGCCTGAACATCTCGGCCCTGATTGTGGGCATCCTCATGACCATCGGATTCATCCTGTTACTGGTGTATGGCTCTGT
C L N I S A L I V G I L M T I G F I L L L V Y G S V

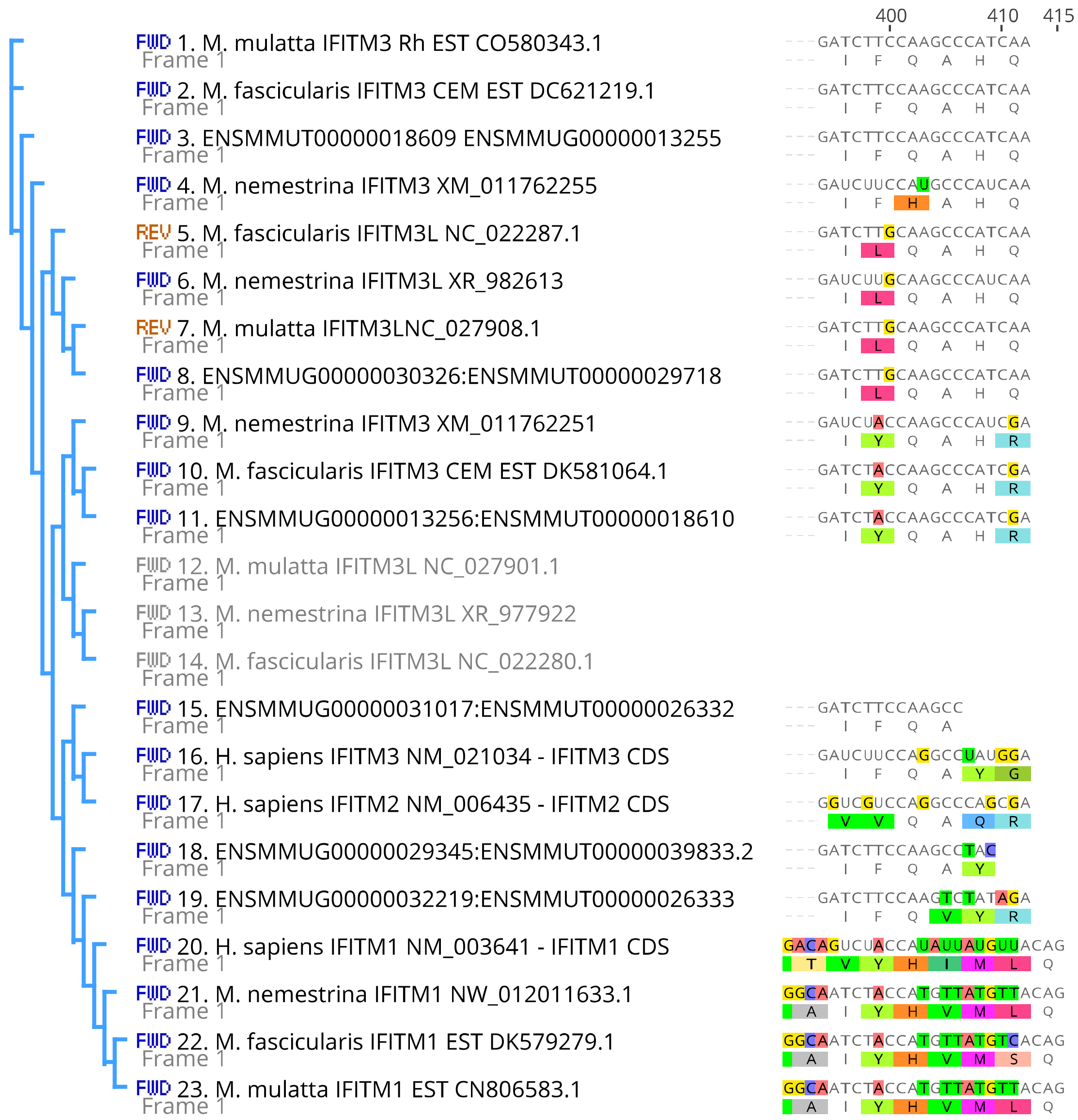
400
410
415
FWD 1. M. mulatta IFITM3 Rh EST CO580343.1
Frame 1
---GATCTTCCAAGCCCATCAA
--- I F Q A H Q
FWD 2. M. fascicularis IFITM3 CEM EST DC621219.1
Frame 1
---GATCTTCCAAGCCCATCAA
--- I F Q A H Q
FWD 3. ENSMMUT00000018609 ENSMMUG00000013255
Frame 1
---GATCTTCCAAGCCCATCAA
--- I F Q A H Q
FWD 4. M. nemestrina IFITM3 XM_011762255
Frame 1
---GAUCUUCCAUGCCCAUCAA
--- I F H A H Q
REV 5. M. fascicularis IFITM3L NC_022287.1
Frame 1
---GATCTTGCAAGCCCATCAA
--- I L Q A H Q
FWD 6. M. nemestrina IFITM3L XR_982613
Frame 1
---GAUCUUGCAAGCCCAUCAA
--- I L Q A H Q
REV 7. M. mulatta IFITM3LNC_027908.1
Frame 1
---GATCTTGCAAGCCCATCAA
--- I L Q A H Q
FWD 8. ENSMMUG00000030326:ENSMMUT00000029718
Frame 1
---GATCTTGCAAGCCCATCAA
--- I L Q A H Q
FWD 9. M. nemestrina IFITM3 XM_011762251
Frame 1
---GAUCUACCAAGCCCAUCGA
--- I Y Q A H R
FWD 10. M. fascicularis IFITM3 CEM EST DK581064.1
Frame 1
---GATCTACCAAGCCCATCGA
--- I Y Q A H R
FWD 11. ENSMMUG00000013256:ENSMMUT00000018610
Frame 1
---GATCTACCAAGCCCATCGA
--- I Y Q A H R
FWD 12. M. mulatta IFITM3L NC_027901.1
Frame 1
FWD 13. M. nemestrina IFITM3L XR_977922
Frame 1
FWD 14. M. fascicularis IFITM3L NC_022280.1
Frame 1
FWD 15. ENSMMUG00000031017:ENSMMUT00000026332
Frame 1
---GATCTTCCAAGCC
--- I F Q A
FWD 16. H. sapiens IFITM3 NM_021034 - IFITM3 CDS
Frame 1
---GAUCUUCCAGGCCUAUGGA
--- I F Q A Y G
FWD 17. H. sapiens IFITM2 NM_006435 - IFITM2 CDS
Frame 1
---GGUCGUCCAGGCCCAGCGA
--- V V Q A Q R
FWD 18. ENSMMUG00000029345:ENSMMUT00000039833.2
Frame 1
---GATCTTCCAAGCCTAC
--- I F Q A Y
FWD 19. ENSMMUG00000032219:ENSMMUT00000026333
Frame 1
---GATCTTCCAAGTCTATAGA
--- I F Q V Y R
FWD 20. H. sapiens IFITM1 NM_003641 - IFITM1 CDS
Frame 1
GACAGUCUACCAUAUUAUGUUACAG
T V Y H I M L Q
FWD 21. M. nemestrina IFITM1 NW_012011633.1
Frame 1
GGCAATCTACCATGTTATGTTACAG
A I Y H V M L Q
FWD 22. M. fascicularis IFITM1 EST DK579279.1
Frame 1
GGCAATCTACCATGTTATGTCACAG
A I Y H V M S Q
FWD 23. M. mulatta IFITM1 EST CN806583.1
Frame 1
GGCAATCTACCATGTTATGTTACAG
A I Y H V M L Q
