# Supplementary material for: Macaque interferon-induced transmembrane proteins limit replication of SHIV strains in an Envelope-dependent manner
Source: PLoS Pathog. 2019 Jul 1;15(7):e1007925. doi: 10.1371/journal.ppat.1007925 (PMC6625738; doi:10.1371/journal.ppat.1007925)
Supplement: S1 Table — (PDF) [file ppat.1007925.s007.pdf]

S1 Table. List of significantly differentially expressed genes.

Upregulated genes:

Symbol	Description	Log fold change
CMPK2	cytidine monophosphate (UMP-CMP) kinase 2, mitochondrial	9.813
MX1	MX dynamin-like GTPase 1	8.523
IFIT1	interferon-induced protein with tetratricopeptide repeats 1	8.069
MX2	MX dynamin-like GTPase 2	7.560
IFIT1B	interferon-induced protein with tetratricopeptide repeats 1B	7.192
IFI27	interferon, alpha-inducible protein 27	6.958
IFIT5	interferon-induced protein with tetratricopeptide repeats 5	5.864
RSAD2	radical S-adenosyl methionine domain containing 2	5.757
HRASLS2	HRAS-like suppressor 2	5.673
USP18	ubiquitin specific peptidase 18	4.631
IFI6	interferon, alpha-inducible protein 6	4.617
IFIT3	interferon-induced protein with tetratricopeptide repeats 3	4.614
IFI44	interferon-induced protein 44	4.250
HERC5	HECT and RLD domain containing E3 ubiquitin protein ligase 5	3.977
IFI44L	interferon-induced protein 44-like	3.803
ISG15	ISG15 ubiquitin-like modifier	3.785
LOC105482948	endogenous retrovirus group FC1 Env polyprotein-like	3.716
XAF1	XIAP associated factor 1	3.434
OASL	2'-5'-oligoadenylate synthetase-like	3.389
HERC6	HECT and RLD domain containing E3 ubiquitin protein ligase family member 6	3.387
IFIT2	interferon-induced protein with tetratricopeptide repeats 2	2.937
DDX58	DEAD (Asp-Glu-Ala-Asp) box polypeptide 58	2.909
DHX58	DEXH (Asp-Glu-X-His) box polypeptide 58	2.845
PLA2G4C	phospholipase A2, group IVC (cytosolic, calcium-independent)	2.737

UBQLNL	ubiquilin-like	2.695
EPSTI1	epithelial stromal interaction 1 (breast)	2.667
IRF7	interferon regulatory factor 7	2.639
LOC105472698	uncharacterized LOC105472698	2.396
LOC105468608	uncharacterized LOC105468608	2.395
BCL2L14	BCL2-like 14 (apoptosis facilitator)	2.312
HELZ2	helicase with zinc finger 2, transcriptional coactivator	2.262
LOC105474583	protein atonal homolog 8-like	2.234
ISG20	interferon stimulated exonuclease gene 20kDa	2.160
HSH2D	hematopoietic SH2 domain containing	1.987
OAS2	2'-5'-oligoadenylate synthetase 2, 69/71kDa	1.966
LOC105494541	uncharacterized LOC105494541	1.858
ODF3B	outer dense fiber of sperm tails 3B	1.778
LOC105465872	YLP motif-containing protein 1-like	1.755
LOC105485135	uncharacterized LOC105485135	1.717
SECTM1	secreted and transmembrane 1	1.686
OAS3	2'-5'-oligoadenylate synthetase 3, 100kDa	1.672
LGALS9	lectin, galactoside-binding, soluble, 9	1.664
LGALS3BP	lectin, galactoside-binding, soluble, 3 binding protein	1.652
EIF2AK2	eukaryotic translation initiation factor 2-alpha kinase 2	1.646
CMTR1	cap methyltransferase 1	1.634
OAS1	2'-5'-oligoadenylate synthetase 1, 40/46kDa	1.629
DDX60	DEAD (Asp-Glu-Ala-Asp) box polypeptide 60	1.628
SLFN14	schlafen family member 14	1.543
AGRN	agrin	1.444
LOC105476888	galectin-9B	1.410
LOC105469170	unconventional myosin-XVB-like	1.390
NT5C3A	5'-nucleotidase, cytosolic IIIA	1.381
TRIM25	tripartite motif containing 25	1.353
LOC105466036	interferon-induced transmembrane protein 3 pseudogene	1.344
TRIM34	tripartite motif containing 34	1.325

IFIH1	interferon induced with helicase C domain 1	1.319
LOC105494124	interferon-induced transmembrane protein 3-like	1.299
LOC105473782	E3 ubiquitin-protein ligase ARIH2-like	1.299
ABHD6	abhydrolase domain containing 6	1.291
CHRNB2	cholinergic receptor, nicotinic, beta 2 (neuronal)	1.261
SCNN1D	sodium channel, non voltage gated 1 delta subunit	1.233
IFI35	interferon-induced protein 35	1.228
TLR7	toll-like receptor 7	1.226
PLSCR1	phospholipid scramblase 1	1.210
RNF213	ring finger protein 213	1.199
SAMD9L	sterile alpha motif domain containing 9-like	1.186
CXCL10	chemokine (C-X-C motif) ligand 10	1.171
LOC105473783	uncharacterized LOC105473783	1.170
TECTA	tectorin alpha	1.165
RTP4	receptor (chemosensory) transporter protein 4	1.155
PML	promyelocytic leukemia	1.150
LOC105466890	translationally-controlled tumor protein pseudogene	1.150
LOC105473984	interferon-induced transmembrane protein 3 pseudogene	1.141
STAT2	signal transducer and activator of transcription 2, 113kDa	1.127
TRIM14	tripartite motif containing 14	1.125
ZNF460	zinc finger protein 460	1.108
LOC105482038	uncharacterized LOC105482038	1.104
TRIM22	tripartite motif containing 22	1.086
LOC105490092	60S ribosomal protein L36a-like	1.079
CXCL11	chemokine (C-X-C motif) ligand 11	1.078
TNK2	tyrosine kinase, non-receptor, 2	1.063
LOC105473954	nuclear body protein SP140	1.059
CACNA1A	calcium channel, voltage-dependent, P/Q type, alpha 1A subunit	1.059
MLKL	mixed lineage kinase domain-like	1.048
LOC105477385	chromosome unknown open reading frame, human C19orf66	1.035
LOC105473997	probable ubiquitin carboxyl-terminal hydrolase creB	1.025

GPC1	glypican 1	1.020
DTX3L	deltex 3 like, E3 ubiquitin ligase	1.015
KCTD14	potassium channel tetramerization domain containing 14	0.982
TNFSF13B	tumor necrosis factor (ligand) superfamily, member 13b	0.974
ABHD1	abhydrolase domain containing 1	0.960
LOC105480733	protein HIRA	0.951
LOC105494127	interferon-induced transmembrane protein 3-like	0.950
ADAR	adenosine deaminase, RNA-specific	0.947
PHF11	PHD finger protein 11	0.943
PEAK1	pseudopodium-enriched atypical kinase 1	0.942
LOC105479241	uncharacterized LOC105479241	0.930
SLFN12L	schlafen family member 12-like	0.919
PARP10	poly (ADP-ribose) polymerase family, member 10	0.913
CCL28	chemokine (C-C motif) ligand 28	0.900
LOC105472067	HLA class I histocompatibility antigen, A-23 alpha chain pseudogene	0.873
UNC93B1	unc-93 homolog B1 (C. elegans)	0.870
IFITM1	interferon induced transmembrane protein 1	0.861
TRANK1	tetratricopeptide repeat and ankyrin repeat containing 1	0.853
PARP9	poly (ADP-ribose) polymerase family, member 9	0.842
TRIM5	tripartite motif containing 5	0.840
POR	P450 (cytochrome) oxidoreductase	0.834
TMEM140	transmembrane protein 140	0.832
DRD4	dopamine receptor D4	0.830
LOC105471721	class I histocompatibility antigen, Gogo-C*0101/C*0102 alpha chain-like	0.830
APOL6	apolipoprotein L, 6	0.816
SP100	SP100 nuclear antigen	0.816
TRIM21	tripartite motif containing 21	0.810
LOC105469854	HLA class I histocompatibility antigen, A-11 alpha chain-like	0.805
SPTLC2	serine palmitoyltransferase, long chain base subunit 2	0.790
FANCB	Fanconi anemia, complementation group B	0.788
PI4K2B	phosphatidylinositol 4-kinase type 2 beta	0.772

SAMD9	sterile alpha motif domain containing 9	0.749
RHBDL3	rhomboid, veinlet-like 3 (Drosophila)	0.743
IFI16	interferon, gamma-inducible protein 16	0.734
SUCNR1	succinate receptor 1	0.719
FBXO6	F-box protein 6	0.713
PNPT1	polyribonucleotide nucleotidyltransferase 1	0.708
IRF9	interferon regulatory factor 9	0.706
CHD7	chromodomain helicase DNA binding protein 7	0.698
MOV10	Mov10 RISC complex RNA helicase	0.664
PARP14	poly (ADP-ribose) polymerase family, member 14	0.664
LOC105493685	caspase-1	0.645
LOC105466242	HLA class I histocompatibility antigen, B-58 alpha chain-like	0.643
LOC105497654	HLA class I histocompatibility antigen, A-30 alpha chain-like	0.635
PARP12	poly (ADP-ribose) polymerase family, member 12	0.633
GBP2	guanylate binding protein 2, interferon-inducible	0.630
FAM26F	family with sequence similarity 26, member F	0.630
REC8	REC8 meiotic recombination protein	0.627
DDX60L	DEAD (Asp-Glu-Ala-Asp) box polypeptide 60-like	0.627
FAM50B	family with sequence similarity 50, member B	0.625
TMEM106A	transmembrane protein 106A	0.623
ZCCHC2	zinc finger, CCHC domain containing 2	0.621
LOC105493179	peptidyl-prolyl cis-trans isomerase A pseudogene	0.618
LOC105494061	metallothionein-2	0.614
LOC105494737	uncharacterized LOC105494737	0.611
LOC105466472	class I histocompatibility antigen, Gogo-B*0103 alpha chain-like	0.608
LOC105467425	uncharacterized LOC105467425	0.605
ATP13A1	ATPase type 13A1	0.594
LOC105478980	histone H4	0.594
DMAP1	DNA methyltransferase 1 associated protein 1	0.591
SMCHD1	structural maintenance of chromosomes flexible hinge domain containing 1	0.588

Downregulated genes:

Symbol	Description	Log fold change
LOC105494138	uncharacterized LOC105494138	-1.573
PFKFB4	6-phosphofructo-2-kinase/fructose-2,6-biphosphatase 4	-1.487
LOC105495202	centrosomal protein of 85 kDa-like	-1.481
TEX14	testis expressed 14	-1.344
CETP	cholesteryl ester transfer protein, plasma	-1.226
PARD6G	par-6 family cell polarity regulator gamma	-1.181
LOC105478049	uncharacterized LOC105478049	-1.169
DPY19L1	dpy-19-like 1 (C. elegans)	-1.054
IL23A	interleukin 23, alpha subunit p19	-0.990
LOC105463640	uncharacterized LOC105463640	-0.925
LOC105485170	high mobility group protein B3 pseudogene	-0.905
LOC105481118	tight junction protein ZO-2-like	-0.903
LOC105468861	uncharacterized LOC105468861	-0.903
LOC105471650	chromosome unknown open reading frame, human C11orf94	-0.883
LOC105488881	60S ribosomal protein L21 pseudogene	-0.855
LOC105464862	cytochrome P450 1B1	-0.835
LOC105487469	uncharacterized LOC105487469	-0.826
LOC105495472	uncharacterized LOC105495472	-0.811
LOC105471002	glucose-6-phosphate 1-dehydrogenase-like	-0.776
LOC105485977	uncharacterized LOC105485977	-0.761
IRAK4	interleukin-1 receptor-associated kinase 4	-0.759
LOC105489019	NKG2D ligand 1-like	-0.750
LOC105467934	Ig heavy chain V-I region V35-like	-0.750
LOC105494912	uncharacterized LOC105494912	-0.722
RPS6KL1	ribosomal protein S6 kinase-like 1	-0.720
LOC105464561	uncharacterized LOC105464561	-0.705
PDK4	pyruvate dehydrogenase kinase, isozyme 4	-0.704

PIP5K1C	phosphatidylinositol-4-phosphate 5-kinase, type I, gamma	-0.702
LOC105466256	putative POTE ankyrin domain family member M	-0.695
ZNF430	zinc finger protein 430	-0.677
IRF4	interferon regulatory factor 4	-0.675
VSIG1	V-set and immunoglobulin domain containing 1	-0.670
CLIP2	CAP-GLY domain containing linker protein 2	-0.670
PABPC3	poly(A) binding protein, cytoplasmic 3	-0.655
ARVCF	armadillo repeat gene deleted in velocardiofacial syndrome	-0.653
EHD3	EH-domain containing 3	-0.643
LOC105495757	interferon alpha/beta receptor 1-like	-0.638
LOC105497049	zinc finger protein 816	-0.632
LOC105481444	eukaryotic translation initiation factor 4B pseudogene	-0.626
SLC25A15	solute carrier family 25 (mitochondrial carrier; ornithine transporter) member 15	-0.625
ACO1	aconitase 1, soluble	-0.620
ABCC4	ATP-binding cassette, sub-family C (CFTR/MRP), member 4	-0.619
ROM1	retinal outer segment membrane protein 1	-0.615
TMEM8B	transmembrane protein 8B	-0.609
MTMR12	myotubularin related protein 12	-0.608
ZC3H7B	zinc finger CCCH-type containing 7B	-0.606
FHL3	four and a half LIM domains 3	-0.604
LOC105468289	60 kDa heat shock protein, mitochondrial-like	-0.604
ZCCHC18	zinc finger, CCHC domain containing 18	-0.590
LOC105499493	40S ribosomal protein S2 pseudogene	-0.587
TUBD1	tubulin, delta 1	-0.586
