# Supplementary material for: Macaque interferon-induced transmembrane proteins limit replication of SHIV strains in an Envelope-dependent manner
Source: PLoS Pathog. 2019 Jul 1;15(7):e1007925. doi: 10.1371/journal.ppat.1007925 (PMC6625738; doi:10.1371/journal.ppat.1007925)
Supplement: S2 Table — (PDF) [file ppat.1007925.s008.pdf]

S2 Table. List of upregulated genes encoding transmembrane helices.

Symbol	Description	RefSeq Protein	Number of predicted helices
CACNA1A	calcium channel, voltage-dependent, P/Q type, alpha 1A subunit	XP_011771175	17
UNC93B1	unc-93 homolog B1 (C. elegans)	XP_011719393	12
RHBDL3	rhomboid, veinlet-like 3 (Drosophila)	XP_011745712	7
ATP13A1	ATPase type 13A1	XP_011752606	7
DRD4	dopamine receptor D4	XP_011760593	7
SUCNR1	succinate receptor 1	XP_011769730	7
FAM26F	family with sequence similarity 26, member F	XP_011712217	4
TMEM140	transmembrane protein 140	XP_011722088	4
ATP13A1	ATPase type 13A1	XP_011751988	4
CHRNB2	cholinergic receptor, nicotinic, beta 2 (neuronal)	XP_011767912	4
APOL6	apolipoprotein L, 6	XP_011710834	3
IFI27	interferon, alpha-inducible protein 27	XP_011715438	2
ABHD1	abhydrolase domain containing 1	XP_011736300	2
LOC105494124	interferon-induced transmembrane protein 3-like	XP_011760553	2
IFITM1	interferon induced transmembrane protein 1	XP_011760555	2
LOC105494127	interferon-induced transmembrane protein 3-like	XP_011760557	2
IFI6	interferon, alpha-inducible protein 6	XP_011761358	2
LOC105466242	HLA class I histocompatibility antigen, B-58 alpha chain-like	XP_011713566	1
SECTM1	secreted and transmembrane 1	XP_011718589	1
HRASLS2	HRAS-like suppressor 2	XP_011718828	1
LOC105471721	class I histocompatibility antigen, Gogo-C*0101/C*0102 alpha chain-like	XP_011722774	1
TMEM106A	transmembrane protein 106A	XP_011723298	1
NT5C3A	5'-nucleotidase, cytosolic IIIA	XP_011729697	1
CXCL10	chemokine (C-X-C motif) ligand 10	NP_001295949	1
CXCL11	chemokine (C-X-C motif) ligand 11	XP_011731993	1
TLR7	toll-like receptor 7	XP_011733444	1

ABHD6	abhydrolase domain containing 6	XP_011741523	1
SLFN12L	schlafen family member 12-like	XP_011745640	1
TNFSF13B	tumor necrosis factor (ligand) superfamily, member 13b	XP_011745904	1
POR	P450 (cytochrome) oxidoreductase	XP_011765625	1
AGRN	agrin	XP_011768613	1
