# Supplementary material for: Macaque interferon-induced transmembrane proteins limit replication of SHIV strains in an Envelope-dependent manner
Source: PLoS Pathog. 2019 Jul 1;15(7):e1007925. doi: 10.1371/journal.ppat.1007925 (PMC6625738; doi:10.1371/journal.ppat.1007925)
Supplement: S3 Table — (PDF) [file ppat.1007925.s009.pdf]

S3 Table. SHIVs used in this study.

SHIV	Source of HIV-1 Envelope	Chronic or Early HIV-1 isolate	Animal Passage	Engineered changes	Reference
SHIV AD8-EO	HIV-1 ADA	Chronic	Serial passage in 5 rhesus macaques followed by coculture in macaque PBMCs	None	[8]
SHIV AD8-OG	HIV-1 ADA	Chronic	None	None	[8]
SHIV SF162P3	HIV-1 SF162	Chronic	Serial passage in 3 rhesus macaques followed by coculture in human PBMCs	None	[6]
SHIV 1157ipd3N4	HIV-1 1157i	Early	Serial passage in 5 rhesus macaques	Additional NF-κB binding site in 3′ LTR	[10]
SHIV Q23AE	HIV-1 Q23	Early	None	A204E point mutation	[25]
SHIV QF495AE	HIV-1 QF495	Early	None	A204E point mutation	[25]
SHIV BG505AE	HIV-1 BG505	Early	None	A204E point mutation	[25]
SHIV MG505GV	HIV-1 MG505	Early	None	G312V point mutation	[25]
